# Primitive Oligomeric RNAs at the Origins of Life on Earth

**DOI:** 10.3390/ijms24032274

**Published:** 2023-01-23

**Authors:** Jacques Demongeot, Michel Thellier

**Affiliations:** 1 Faculty of Medicine, Université Grenoble Alpes, Laboratory AGEIS EA 7407 Tools for e-Gnosis Medical, 38700 Grenoble, France; 2 Académie des Sciences, Section Biologie Integrative, 75006 Paris, France

**Keywords:** ring world, origin of life, ancient ribosomal proteins, small RNAs

## Abstract

There are several theories on the origin of life, which differ by choosing the preponderant factor of emergence: main function (autocatalysis versus replication), initial location (black smokers versus ponds) or first molecule (RNA versus DNA). Among the two last ones, the first assumes that an RNA world involving a collaboration of small RNAs with amino-acids pre-existed and the second that DNA–enzyme–lipid complexes existed first. The debate between these classic theories is not closed and the arguments for one or the other of these theories have recently fueled a debate in which the two have a high degree of likelihood. It therefore seems interesting to propose a third intermediate way, based on the existence of an RNA that may have existed before the latter stages postulated by these theories, and therefore may be the missing link towards a common origin of them. To search for a possible ancestral structure, we propose as candidate a small RNA existing in ring or hairpin form in the early stages of life, which could have acted as a “proto-ribosome” by favoring the synthesis of the first peptides. Remnants of this putative candidate RNA exist in molecules nowadays involved in the ribosomal factory, the concentrations of these relics depending on the seniority of these molecules within the translation process.

## 1. Introduction

When the Latin poet Ovid was writing about metamorphoses two thousand years ago, things were very simple: living beings were spontaneously generated from non-living matter [1]. It was relatively recently, following Francesco Redi [2], Lazzaro Spallanzani [3], Louis Pasteur [4] and many others that the belief in spontaneous generation was finally abandoned. From that time, the statement that life on Earth can be born only from preexisting living organisms was admitted as a rule by almost everybody. Moreover, although the lichen *Xanthoria elegans* remained viable after 18 months of space exposure outside the International Space Station (ISS) [5], it is extremely unlikely that life came to Earth from another planet (which would imply travelling within the cosmic vacuum for thousands, if not millions or billions of years). The logical conclusion is that there was an exception to the rule, i.e., that at least once life emerged on Earth from non-living precursors.

Several theories have dealt with the problem of the origin of life. They differ in focusing each on a preponderant factor of emergence: the main primitive function (autocatalysis versus replication), the initial location of the first living systems (black smokers versus ponds) or the first molecule involved in the origin of life (RNA versus DNA). In the last couple of opposite theories, the first assumes that a primitive “RNA world” has existed. It was based on the collaboration of nucleic and amino-acids polymers, the organic synthesis of which is obtained in absence of any living process [6,7,8,9,10,11,12,13,14,15,16]. The RNA world hypothesis was proposed in the 1960s. Miller’s experiment had shown that a spontaneous production of nucleotides and amino-acids was possible under the primitive Earth conditions assumed to exist at that time, especially in the vicinity of intra-ocean hot springs [6,14]. Two decades later, it was admitted that this was the most likely explanation for how life started, because a lot of experimental and theoretical works had shown it was possible that due to the collaboration between small RNAs and amino-acids, primitive self-replicating systems could survive. An example of structures capable of self-replication, elongation and circularization is provided by small ribozymes [17], e.g., the *Azoarcus* ribozyme [18,19,20], but RNA self-replication based on a replicase ribozyme has never been demonstrated and is difficult to achieve.

In the second theory, it was assumed that a primitive DNA–enzyme–lipid complex pre-existed, which allowed storage of information in DNA, replication by polymerases and individualization by lipids of a living machine, which were then subject to Darwinian selection. The main criticism about the RNA world was that it should contain reflexive information together with information, i.e., information which, decoded by the system, produces the components that perform exactly that particular decoding. Moreover, it was proposed that protein-like molecules were better candidates than RNA for having been the first self-replicators functioning on the planet.

To solve this problem, authors recently used, in the spirit of Stanley Miller’s pioneering experiment, a hydrogen cyanide-based chemical medium mimicking early Earth conditions to synthesize the four purine and pyrimidine bases [21,22]: the first two, the purine bases deoxyadenosine and deoxyinosine, were in their DNA deoxy form, the other two, the pyrimidine bases Cytidine and Uridine, having their form observed in RNA. The conclusion was that both deoxyribonucleosides and ribonucleosides may have coexisted before the emergence of life. For example, during the Late Heavy Bombardment (LHB) which occurred on Earth about 4.1–3.8 billions of years ago, hydrogen cyanide could be the starting compound, then all the RNA canonical nucleobases and the simplest amino acids could appear [23,24,25].

More recently, it was suggested to integrate the two theories with each other. DNA, enzymes and lipids were assumed to have interfered early with the engineering of the primitive RNAs [26] in order to give it a selective advantage over purely chemical systems [27,28,29,30,31,32,33,34,35,36]. This interpretation combines (i) the existence of a form of storage within primitive RNAs, also making it possible to promote peptide synthesis and the appearance of proto-genes consisting of combinations of fragments of primitive RNAs, (ii) the random inclusion of new RNA materials giving birth progressively to proteins ensuring biosynthesis and replication of DNA, (iii) the formation of lipid membranes compartmentalizing the spatial location of the functions (i) and (ii).

We will present successively, in Section 2, the methodology used for searching the remnants of the first hypothetical small RNA molecules. In Section 3, we present the results of such an investigation, and in Section 4, we discuss the relevance and validity of the results described in the previous Sections.

## 2. Results

We start by searching in the oldest living organisms, the Archaea, the most conserved sequences in the machinery devoted to the protein building, that is, the ribosome and associated molecules as tRNAs, amino-acyl tRNA ligases, nucleolin, etc. Concerning the ribosome, we will examine, namely, its smallest component, the 5S rRNA as well as the most ancient ribosomal RNAs and proteins following the classification by authors such as G. Caetano-Anollés [37,38,39,40,41,42,43,44,45]. For example, in [45], the most likely sub-sequences in 500 archaeal tRNAs are the following: 5′-UGGU-3′ for the tRNA D-loop, 5′-UUCAA-3′ for the T-loop and 5′-CUGCCA-3′ for the Gly-tRNA^GCC^ AC-loop.

**Figure 1:**
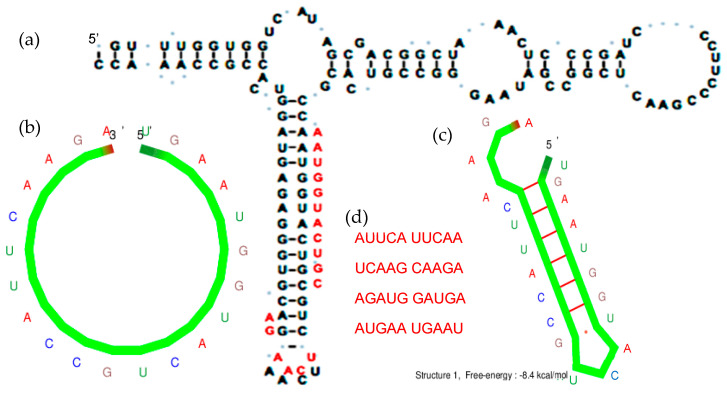
(**a**) AL hemi-sequence AAUGGUACUGC (in red) and AL-hexamer UCAAGA (in red) in a stem of the 5S rRNA of *Rhodobacter sphaeroides* [38]. (**b**) Ring form of AL. (**c**) Optimal hairpin form of AL (obtained using Kinefold^®^ [47]). (**d**) Most likely pentamers observed after denaturation of the hairpin form of AL, called most plausible observed pentamers or p-pentamers.

If we search similar sequences among the 5S rRNAs [46], we find the frequent sequence 5′-AAUGGUACUGC-3′ (see Figure 1a and Supplementary Materials). By complementing the beginning AAUGGUA of this sequence, we obtain the sequence UGCCAUU, and by searching the shortest and most stable hairpin with a 9-nt long stem containing these sequences, we obtain by using the software Kinefold^®^ [47] the hairpin head AGA, hence, a 22-nt sequence, we called AL [28,29,30,31] (for Archetypal loop, because it contains the loops of archaeal tRNAs): UGAAUGGUA/C/UGCCAUUCA/AGA.

If we consider the AL sequence as a source of primitive information on the genetic code [48,49,50], it presents a high level of optimality by storing in minimal length a maximum of biologically relevant information. The genetic information-based properties of AL as top-down clues supporting both (i) a common evolutionary conserved origin from currently observed sequences and (ii) a coherence with genetic code can be summarized:

- The subsequences of AL are the most frequent in 5S rRNAs and in Gly-tRNAs.
- All dinucleotides appear at least once (except CG, because of CG suppression).
- Among the rings satisfying the minimality principle “to be as short as possible and contain at least one codon of each amino acid class of synonymy” of the genetic code (Figure 1b), there is no solution for a length below 22 nucleotides. For length 22, there are 29,520 solutions (among 16 10^12^ of possible solutions) containing only one codon AUN repeated, N being G in 52% of the cases.
- From these 29,520 solutions, the search by Kinefold^®^ for the most stable hairpins gave 25 rings of length 9, with a head of length 3, the most stable being AGA (Figure 1c).
- From these 25 rings, 19 encompass both a start and a stop codon.
- Through the calculation of several distances (e.g., circular Hamming distance, permutation distance and edit distance), one singular ring (the AL ring) exhibits a minimum average distance as compared to the others. Therefore, only this sequence can be that acting as a barycenter of the set of the 18 others.

Among its pentamer subsequences, we have selected the eight pentamers resulting from the opening of its hairpin head conformation (Figure 1c), i.e., the set P = {ATTCA TTCAA TCAAG CAAGA AGATG GATGA ATGAA TGAAT}. P is the set of the most likely pentamers observed after denaturation of the hairpin form of AL, called most plausibly observed pentamers or p-pentamers, and we use in the following, as a proximity criterion to AL of a nucleotide sequence, its relative number either of all its pentamers or only of the eight p-pentamers. In other words, the AL-proximity of a sequence is the percentage of pentamers common to AL and this sequence, equal in random case to 2.15 ± 0.75% (95%-confidence interval) for a 1000-nt length sequence.

The ancestral molecules that participated in the first prebiotic assemblies might have been RNA oligomers and small peptides formed from nucleotides on the one hand and amino-acids on the other hand, both being present in hot springs and/or in clay matrices [51]. We may consider that hot springs and clays were sort of physicochemical “marriage agencies” favoring the amino-acid/nucleotide interactions necessary for the synthesis of new peptides and new nucleic acids. It was also wondered [52] about the possibility that the violent processes occurring on the nascent Earth (4.6 billion years ago) were the starting point for the transformations that were to lead to the appearance of life on Earth. At that time, the Earth was far too hot for organic molecules to have existed on it, and these processes would therefore have involved at first only mineral substances. Then, they could have evolved during the slow cooling of the Earth towards the progressive involvement of organic compounds, thus joining the paths envisaged by the classic theories (“RNA first” or “DNA first”).

**Figure 2:**
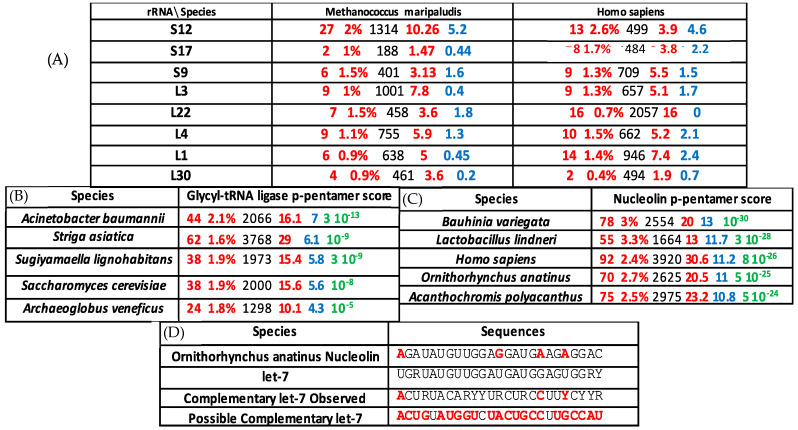
(**A**) Some of the ribosomal protein genes and RNAs in Caetano-Anollés’ order [33] with successive indications of the observed number P_o_ of p-pentamers common with AL in their nucleotide sequence (red), their percentage inside the set of tall heir pentamers (red), their sequence size (black), the expected number P_e_ (red), and the number of standard deviations between P_o_ and P_e_ (blue). (**B**) Same representation as in (**A**) for the Glycyl-tRNA ligase of various species, plus the probability to observe such P_o_’s (green). (**C**) Same representation as in (**B**) for the nucleolin of various species. (**D**) Sub-sequence of the nucleolin gene of *Ornithorhynchus anatinus* matching with its mRNA inhibitor, the microRNA let7, whose complementary sequence contains parts from AL sequence (in red) (cf. Figure S1 in Supplementary Materials).

**Figure 3:**
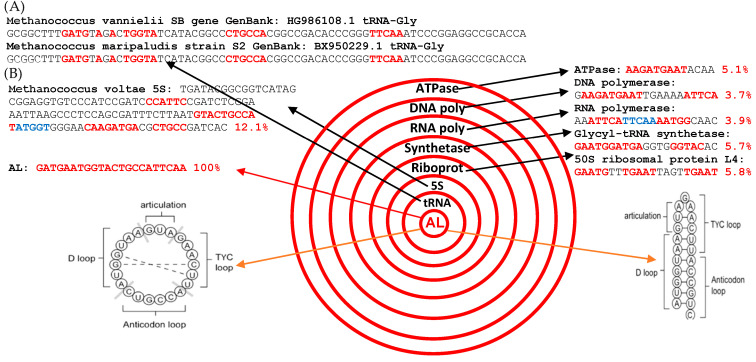
(**A**) Primary structure of Gly-tRNA in *Methanococcus vanniellii or Maripaludis voltae* showing the consecutive subsequences of the AL-ring inside the successive tRNA loops. (**B**) AL-proximity of the main RNA or protein molecules participating to the ribosomal translation in *Methanococcus voltae*. The expected percentage is 2.15 ± 0.75% (95%-confidence interval) for a 1000-nt length sequence (cf. Supplementary Materials). The pentamers are indicated in red or in blue in presence of overlapping.

The small RNAs would be good candidates to be actors and witness of this evolution from a purely mineral behavior to the organic world. If we consider that the current most effective machinery for protein synthesis is the ribosome, we may wonder whether a “memory” of the primitive machinery possibly exists in that particle. Such a memory should be almost universal, i.e., it should be found from the origin in a large majority of ribosomal mechanisms of different species, which is the case in Figure 2, where the p-pentamer score is calculated by counting the number of expected standard deviation between observed and expected numbers of p-pentamers (the most probable observed pentamers after denaturation of the AL hairpin), called the p-pentamer score. Remnants of AL in genes involved in the protein translation machinery can be placed on concentric circles whose diameter order corresponds to the order of the AL-proximities. This last order respects both species seniority and evolutionary appearance order of the molecules involved in protein translation (Figure 3).

The constraint of evolutionary seniority and interspecific invariance leads us to systematically examine the most robust invariants, both in the vertical dimension linked to evolution and in the horizontal dimension linked to speciation. A realistic candidate for the ancestral molecules could be a primitive RNA similar to the succession of the nucleotides contained in the loops of a large majority of tRNAs as observed at present [29,30], especially those of Archaea such as *Methanococcus vanniellii or Maripaludis voltae* (cf. Figure 3 and Tables S1–S7 in Supplementary Materials) or of their mitochondria (whose the most plausible ancestor, *Rickettsia prowazekii*, has all the characteristics of an ancient organism [30,31]): AL meets the criteria of such a realistic candidate.

## 3. Material and Methods

All the material is coming from the public databases GtRNAdb [39] and 5S RNAdb [46]. We have used for obtaining the most stable hairpin structures the online facilities of the software Kinefold^®^ [47].

## 4. Discussion

Less than a decade later, Stanley Miller [6] announced the production of simple organic molecules under atmospheric conditions resembling those assumed at that time to have existed on the primitive Earth. At the end of the 20th century, much skill had already been devoted to clear up the stages by which the cell structure and the coded macromolecules typical of today’s living beings were acquired. Freeman Dyson’s vision [53], that two dynamic processes, metabolism and replication, were essential to discriminate living organisms from inanimate matter has prompted researchers to follow an experimental approach. Cold Spring Harbor Laboratory Press has gathered together articles by specialists of various origins sharing a common interest in the origins of life on Earth with a focus on the evolution of the ribosome [54,55,56]. The review articles by Jason P. Schrum, Ting F. Zhu and Jack W. Szostak [57,58] provide an elaborate analysis of the efforts to devise a laboratory protocell model, with the aim to evaluate a pathway for the transition from complex prebiotic chemistry to simple biology.

Although this ambitious goal has still not been achieved, efforts are continuing, eliminating step by step the remaining gaps in the knowledge of prebiotic chemistry. One step could be represented by the present effort to solve the challenge of the origins, DNA first or RNA first. The ring AL we have exhibited could be an element of an answer, because its purely informative, combinatorial and chemical properties, could help to: (i) build the first peptides by promoting the coming together of amino acids synthesized in the primitive atmosphere (as demonstrated by S. Miller), thus acting as a proto-ribosome. These amino acids could have created more stable peptide bonds between them than their weak electromagnetic bonds to AL and then, (ii) promote a primitive metabolism from these peptides, the first role of which would have been to promote the replication of AL and its spatial individuation by organizing a first water-repellent peptide or lipid–peptide structure serving as a proto-membrane (as postulated by Dyson [53]).

The major criticisms that can be made to our approach are three-fold: (i) the proposed structure is too small in size to be functional, but recent works show that very small RNAs of size 12 nt can have an important function, such as being able to modulate the microRNA from which they derive [59], (ii) the experimental testing of the creation of peptide structures by small RNAs can be performed [60,61], and it has already been carried out in the case of two amino acids on an RNA template [62,63] and could be generalized in the near future, and (iii) the proteins conformations alone could be at the origin of the fitness ruling the first steps of evolution, but they are also concerned by the underlying information of the nucleotide sequence of their genes, which allows the choice among sequences having similar shape abilities of enzymatic catalysis for their substrates. The sequence which imposes itself among those realizing a given functional form could the one which resembles the original sequences. The final protein fitness is needed, of course, to perform an enzymatic function, but when it can be performed by different sequences (e.g., when in well-adapted species with same functions and different genotypes), those which are observable, especially among old species and functions, can have kept a close relationship (in terms of high statistical significance of common occurrence of small sequences such as pentamers or hexamers [37]) with putative initial sequences such as AL (Figure 2 and Figure 3).

## 5. Conclusions and Perspectives

If the birth of life really took place shortly after Earth’s creation, it is likely that it will remain not-understood forever. It might have been anything, perhaps a very improbable event occurring in an extremely brief instant, such as the Late Heavy Bombardment (LHB) on Earth. However, even in that case, it is a goal of major interest to continue deciphering the history of life on Earth, as close as possible to the non-understandable discontinuity “non-living/living”. New data can be gathered in different ways to check the theories and the present proposal of a primitive ring/hairpin RNA structure spontaneously generated among random sequences and the shortest to present a great coherence with the genetic code could help the theoretical and experimental understanding of this primordial founding event of life on Earth. Concerning the experimental validation, we have proposed in [60] a test in vitro to check that (i) amino-acids “prefer” their corresponding nucleotide triplets (their affinity being better for the latter than for others codons), and (ii) two amino-acids can be located together on the sequence of two adjacent codons from their class of synonymy. The difficulty comes from the very weak expected interactions. So as to limit the influence of other competitive interactions, no cross or self-interactions must be allowed between hexanucleotides. Affinity chromatography techniques are not well-suited, but it could be possible to measure the small chemical equilibrium shifts that should occur due to the weak association between specific amino-acids and their codons followed by the constitution of a dipeptide. A good manner to study them could be to use different RNA ring preparations in solution or fixed on a support, one containing AL and the others a random RNA ring of length 22, plus amino-acids, in order to detect a difference in the final dipeptide concentration, the ring AL favoring the dipeptide synthesis.

## Data Availability

Not applicable.

## Supplementary Materials

The following supporting information can be downloaded at: https://www.mdpi.com/article/10.3390/ijms24032274/s1, Figure S1: MicroRNA let7 secondary structure; Table S1: tRNA-GlyGCC of 70 Archaea; Table S2: tRNAs from various species; Table S3: Percentage of AL-pentamers in Methanococcus voltae genes coding for proteins involved in ribosomal translation; Table S4: rRNAs and genes coding for rproteins from different species; Table S5: Glycyl-tRNA ligase sequences from different species; Table S6: Nucleolin sequences from different species; Table S7: 5S of Archaea with their AL-pentamers.

## References

[1] Ovidius P. (1524). P. Ovidii Nasonis Metamorphosis Libri Moralizati cum Pulcherrimum Fabularum Principalium Figuris, Liber XV.

[2] Redi F. (1668). Esperienze Intorno alla Generazione Degl’insetti.

[3] Spallanzani L. (1787). Opuscules de Physique Animale et Végétale.

[4] Pasteur L. (1907). Un Débat Scientifique Pouchet et Pasteur, 1858–1868.

[5] Brandt A., De Vera J.P., Onofri S., Ott S. (2015). Viability of the lichen Xanthoria elegans and its symbionts after 18 months of space exposure and simulated Mars conditions on the ISS. Int. J. Astrobiol..

[6] Miller S.L. (1953). A Production of amino acids under possible primitive Earth conditions. Science.

[7] Woese C. (1967). The Genetic Code.

[8] Paecht-Horowitz M., Berger J., Katchalsky A. (1970). Prebiotic synthesis of polypeptides by heterogeneous polycondensation of amino-acid adenylates. Nature.

[9] Eigen M. (1971). Selforganization of matter and the evolution of biological macromolecules. Naturwissenschaften.

[10] Cox R.A., Katchalsky A. (1972). Hysteresis and conformational changes in ribosomal ribonucleic acid. Biochem. J..

[11] Eigen M., Winkler-Oswatitsch R. (1981). Transfer-RNA: The early adaptor. Naturwissenschaften.

[12] Gilbert W. (1986). Origin of life: The RNA world. Nature.

[13] Eigen M. (1993). The origin of genetic information: Viruses as models. Gene.

[14] Hobish M.K., Wickramasinghe N.S.M.D., Ponnamperuma C. (1995). Direct interaction between amino-acids and nucleotides as a possible physico-chemical basis for the origin of the genetic code. Adv. Space Res..

[15] Tamura K., Schimmel P. (2001). Oligonucleotide-directed peptide synthesis in a ribosome- and ribozyme-free system. Proc. Natl. Acad. Sci. USA.

[16] Bada J.L., Lazcano A. (2003). Prebiotic soup—Revisiting the Miller experiment. Science.

[17] Vauléon S., Müller S. (2003). External Regulation of Hairpin Ribozyme Activity by an Oligonucleotide Effector. ChemBioChem.

[18] Kuo L.Y., Davidson L.A., Pico S. (1999). Characterization of the Azoarcus ribozyme: Tight binding to guanosine and substrate by an unusually small group I ribozyme. BBA.

[19] Jayathilaka T.S., Lehman N. (2018). Spontaneous Covalent Self-Assembly of the Azoarcus Ribozyme from Five Fragments. ChemBioChem.

[20] Jeancolas C., Matsubara Y.J., Vybornyi M., Lambert C.N., Blokhuis A., Alline T., Griffiths A.D., Sandeep A., Sandeep K., Nghe P. (2021). RNA diversification by a self-reproducing ribozyme revealed by deep sequencing and kinetic modelling. Chem. Commun..

[21] Tjhung K.F., Shokhirev M.N., Horning D.P., Joyce G.F. (2020). An RNA polymerase ribozyme that synthesizes its own ancestor. Proc. Natl. Acad. Sci. USA.

[22] Xu J., Chmela V., Green N.J., Russell D.A., Janicki M.J., Góra R.W., Szabla R., Bond A.D., Sutherland J.D. (2020). Selective prebiotic formation of RNA pyrimidine and DNA purine nucleosides. Nature.

[23] Hörst S.M., Yelle R.V., Buch A., Carrasco N., Cernogora G., Dutuit O., Quirico E., Sciamma-O’Brien E., Smith M.A., Somogyi Á. (2012). Formation of Amino Acids and Nucleotide Bases in a Titan Atmosphere Simulation Experiment. Astrobiology.

[24] Ferus M., Rimmer P., Cassone G., Knížek A., Civiš S., Šponer J.E., Ivanek O., Šponer J., Saeidfirozeh H., Kubelík P. (2020). One-Pot Hydrogen Cyanide-Based Prebiotic Synthesis of Canonical Nucleobases and Glycine Initiated by High-Velocity Impacts on Early Earth. Astrobiology.

[25] Ferus M., Knížek A., Petera L., Pastorek A., Hrnčířová J., Jankovič L., Ivanek O., Šponer J., Křivková A., Saeidfirozeh H. (2022). Formamide-Based Post-impact Thermal Prebiotic Synthesis in Simulated Craters: Intermediates, Products and Mechanism. Front. Astron. Space Sci..

[26] Xu J., Green N.J., Russell D.A., Liu Z., Sutherland J.D. (2021). Prebiotic Photochemical Coproduction of Purine Ribo- and Deoxyribonucleosides. J. Am. Chem. Soc..

[27] Kristoffersen E.L., Burman M., Noy A., Holliger P. (2022). Rolling circle RNA synthesis catalyzed by RNA. Elife.

[28] Demongeot J. (1975). Au Sujet de Quelques Modèles Stochastiques Appliqués à la Biologie. Modélisation et Simulation.

[29] Demongeot J. (1978). Sur la possibilité de considérer le code génétique comme un code à enchaînement. Rev. De Biomaths.

[30] Demongeot J., Besson J. (1983). Code génétique et codes à enchaînement I. C. R. Acad. Sc. Série III.

[31] Demongeot J., Moreira A. (2007). A circular RNA at the origin of life. JTB.

[32] Kauffman S.A. (2011). Approaches to the origin of life on Earth. Life.

[33] Higgs P., Lehman N. (2015). The RNA World: Molecular cooperation at the origins of life. Nat. Rev. Genet..

[34] Demongeot J., Norris V. (2019). Emergence of a “Cyclosome” in a primitive network capable of building “infinite” proteins. Life.

[35] Demongeot J., Moreira A., Seligmann H. (2021). Negative CG dinucleotide bias: An explanation based on feedback loops between Arginine codon assignments and theoretical minimal RNA rings. Bioessays.

[36] Norris V., Demongeot J. (2022). The Ring World: Eversion of small double-stranded polynucleotide circlets at the origin of DNA double helix, RNA polymerization, triplet code, twenty amino acids, and strand asymmetry. IJMS.

[37] Root-Bernstein R., Kim Y., Sanjay A., Burton Z.F. (2016). tRNA evolution from the proto-tRNA minihelix world. Transcription.

[38] Kim S.H., Suddath F.L., Quigley G.J., McPherson A., Sussman J.L., Wang A.H.J., Seeman N.C., Rich A. (1974). Three-dimensional tertiary structure of yeast phenylalanine transfer RNA. Science.

[39] GtRNAdb. http://lowelab.ucsc.edu/GtRNAdb/.

[40] Gray M. (1998). Rickettsia, typhus and the mitochondrial connection. Nature.

[41] Harish A., Caetano-Anollés G. (2012). Ribosomal history reveals origins of modern protein synthesis. PLoS ONE.

[42] Gospodinov A., Kunnev D. (2020). Universal Codons with Enrichment from GC to AU Nucleotide Composition Reveal a Chronological Assignment from Early to Late Along with LUCA Formation. Life.

[43] Pak D., Root-Bernstein R., Burton Z.F. (2017). tRNA structure and evolution and standardization to the three nucleotides genetic code. Transcription.

[44] Kim Y., Opron K., Burton Z.F. (2019). A tRNA- and Anticodon-Centric View of the Evolution of Aminoacyl-tRNA Synthetases, tRNAomes, and the Genetic Code. Life.

[45] Demongeot J., Seligmann H. (2022). Evolution of tRNA subelement accretion from small and large ribosomal RNAs. Biosystems.

[46] 5S RNAdb. http://www.combio.pl/rrna/alignment/.

[47] Kinefold. http://kinefold.curie.fr.

[48] Demongeot J., Seligmann H. (2019). Theoretical minimal RNA rings recapitulate the order of the genetic code’s codon-amino acid assignments. JTB.

[49] Demongeot J., Seligmann H. (2019). Spontaneous evolution of circular codes in theoretical minimal RNA rings. Gene.

[50] Demongeot J., Seligmann H. (2019). The Uroboros theory of life’s origin: 22-nucleotide theoretical minimal RNA rings reflect evolution of genetic code and tRNA-rRNA translation machineries. Acta Biotheor..

[51] Müller F., Escobar L., Xu F., Węgrzyn E., Nainytė M., Amatov T., Chan C.Y., Pichler A., Carell T. (2022). A prebiotically plausible scenario of an RNA–peptide world. Nature.

[52] Thellier M. (2023). Origins of life: Proposal for an alternative approach. Progress Bot..

[53] Dyson F. (1999). Origins of Life.

[54] Fox G.E. (2010). Origin and Evolution of the Ribosome. Cold Spring Harb. Perspect. Biol..

[55] Schrum J.P., Zhu T.F., Szostak J.W. (2010). The origins of cellular life. Cold Spring Harb. Perspect. Biol..

[56] Cech T.R., Steitz J.A., Atkins J.F. (2019). RNA Worlds: New Tools for Deep Exploration.

[57] Blain J.C., Szostrak J.W. (2014). Progress towards synthetic cells. Ann. Rev. Biochem..

[58] Ding D., Zhou L., Giurgiu C., Szostak J.W. (2022). Kinetic explanation for the sequence biases observed in the nonenzymatic copying of RNA templates. Nucleic Acids Res..

[59] Diallo I., Ho J., Lalaouna D., Massé E., Provost P. (2022). RNA Sequencing Unveils Very Small RNAs with Potential Regulatory Functions in Bacteria. Front. Mol. Biosci..

[60] Demongeot J., Glade N., Moreira A., Vial L. (2009). RNA relics and origin of life. Int. J. Mol. Sci..

[61] Demongeot J., Henrion-Caude A. (2020). T Footprints of a Singular 22-Nucleotide RNA Ring at the Origin of Life. Biology.

[62] Tamura K., Schimmel P.R. (2003). Peptide synthesis with a template-like RNA guide and aminoacyl phosphate adaptors. Proc. Natl. Acad. Sci. USA.

[63] Tamura K., Schimmel P.R. (2006). Chiral-selective aminoacylation of an RNA minihelix: Mechanistic features and chiral suppression. Proc. Natl. Acad. Sci. USA.

