# Supplementary material for: Primitive Oligomeric RNAs at the Origins of Life on Earth
Source: Int J Mol Sci. 2023 Jan 23;24(3):2274. doi: 10.3390/ijms24032274 (PMC9916791; doi:10.3390/ijms24032274)
Supplement: Supplementary file 1 [file ijms-24-02274-s001.zip › ijms-2136505-supplementary.pdf]

Table S1: tRNA-Gly^{GCC} of 70 Archaea, whose number of hexamers CTGCCA observed is 37 and expected 70x70/46 = 1.2±1.8 (95% CI)

Candidatus Korarchaeum cryptofilum OPF8, complete sequence NCBI Reference Sequence: NC_010482.1: c1321063-1320990 tRNA-Gly

GCGGCCGTAGTCTAGCC**TGG**ACAGGATGGGGGC**CTGCCA**CGTCCCAGACCCGGG**TTCAA**ATCCCGGCGGCCGCA

Thermofilum pendens Hrk 5, complete sequence NCBI Reference S equence: NC_008698.1: 796113-796158,796179-796209 tRNA-Gly

GCGGCCGTAGTCTAGTC**TGGTA**GGATGGCGGC**CTGCCA**CGCCGCAGAACCCGGG**TTCAA**ATCCCGGCGGCCGCACCA

Caldivirga maquilingensis tRNA-Gly (GCC), complete sequence, strain: IC-167 GenBank: AB470218.1 tRNA-Gly

GCGGCCGTAGTCTAGTC**TGGT**TTAGGATGGCGGC**CTGCC**GCGCCGCAGATCCCGGG**TTCAA**ATCCCGGCGGCCGCA

Vulcanisaeta distributa DSM 14429, complete genome GenBank: CP002100.1: 953496-953593 tRNA-Gly

GGGCCCGTAGTCTAGCC**TGGTA**GGATGCCCAGGGGCCGA**TGCC**CCGTACAAGCCTGACGCGCGGGAGATC
CCGGG**TTCAAG**TCCCGGCGGGCCCACCA

Thermoproteus tenax Kra 1, complete genome GenBank: FN869859.1: c40937-40818 tRNA-Gly

GCGGCGGTAGTCTAGCC**TGGT**TAGGGCGGCCAAAAGCCCCCAGAGTAGGATGGCGGC**CTGCCA**GGCCGCA
CCATCGGCGCAGTGAGCCGTTGATCCCGGG**TTCAA**ATCCCGGCCGCCGCA

Pyrobaculum neutrophilum V24Sta, NCBI Reference Sequence: NC_010525.1: 1441482-1441503,1441534-1441590 tRNA-Gly

GCGGCGGTAGTCTAGCC**TGGT**TTAGGATGGCGGC**CTGCCA**AGCCGTTGATCCCGGG**TTCAA**ATCCCGGCC
GCCGCACCA

Pyrobaculum arsenaticum DSM 13514, complete sequence NCBI Reference Sequence: NC_009376.1: c1479953-1479930,c1479895-1479841 tRNA-Gly

GCGGCGGTAGTCTAGCC**TGGT**TTAGGATGGCGGC**CTGCCA**AGCCGTTGATCCCGGG**TTCAA**ATCCCGGCCGCCGCACCA

Pyrobaculum aerophilum str. IM2, complete sequence NCBI Reference Sequence: NC_003364.1: 1904041-1904119 tRNA-Gly

GCGGCGGTAGTCTAGCC**TGGT**TTAGGATGGCGGC**CTGCCA**AGCCGTTGATCCCGGG**TTCAA**ATCCCGGCCGCCGCACCA

Sulfolobus tokodaii str. 7 DNA, complete genome GenBank: BA000023.2: 1719268-1719343 tRNA-Gly

GCGGCCGTAGTCTAGCC**TGG**ATTAGGACGCCGGC**CTGCCA**CGCCGGAGGTCCCGGG**TTCAA**ATCCCGGCGGTCGCA

Sulfolobus solfataricus strain P1 genome assembly, chromosome: I GenBank: LT549890.1: 388180-388255 tRNA-Gly

GCGGCCGTAGTCTAGCC**TGG**ATTAGGACGCCTGC**CTGCCA**CGCAGGAGGTCCCGGG**TTCAA**ATCCCGGCGGCCGC

Metallosphaera yellowstonensis MK1 genomic scaffold MetMK1scaffold_19, whole genome shotgun sequence GenBank: JH597770.1: 457522-457597 tRNA-Gly

GCGGCCGTAGTCTAGCC**TGG**ATTAGGACGCCTGC**CTGCCA**CGCAGGAGGTCCCGGG**TTCAA**ATCCCGGCGGTCGCA

Metallosphaera cuprina Ar-4, complete sequence NCBI Reference Sequence: NC_015435.1: 110857-110932 tRNA-Gly

GCGGCCGTAGTCTAGCC**TGG**ATTAGGACGCCTGC**CTGCCA**CGCAGGAGGTCCCGGG**TTCAA**ATCCCGGCGGTCGCA

Ignicoccus hospitalis KIN4/I, complete sequence NCBI Reference Sequence: NC_009776.1: 289795-289873 tRNA-Gly

GCGGCGGTCGTCTAGCC**TGG**ACTAGGACGCCGGC**CTGCCA**CGCCGGAGATCCCGGGTTC**AA**ATCCCGGCCGCCGCACCA

Hyperthermus butylicus DSM 5456, complete sequence NCBI Reference Sequence: NC_008818.1: c376263-376186 tRNA-Gly

GCGGCGGTCGTCTAGCC**TGG**ACTAGGACGCCGGC**C**C**CCCA**AGCCGGTCACCCGGG**TTCAA**ATCCCGGCCGCCGCACCA

Aeropyrum pernix K1, complete sequence NCBI Reference Sequence: NC_000854.2: c467913-467835 tRNA-Gly

GCGGCGGTCGTCTAGCC**TGG**ACTAGGACGCCGGC**CTGCCAA**GCCGGAGGTCCCGGG**TTCAA**ATCCCGGCCGCCGCACCA

Ignisphaera aggregans DSM 17230, complete genome GenBank: CP002098.1: c1027071-1026993 tRNA-Gly

GCGGCGGTCGTCTAGCC**TGGT**CTAGGACGCCGGC**CTGCCA**AGCCGGAGATCCCGGG**TTCAA**ATCCCGGCCGCCGCACCA

Staphylothermus marinus F1, complete sequence NCBI Reference Sequence: NC_009033.1: 624851-624890,624916-624954 tRNA-Gly

GCGGCGGTCGTCTAGCC**TGG**ACTAGGACGCCGGC**CTGCCA**CGCCGGAGATCCCGGG**TTCAA**ATCCCGGCCGCCGCACCA

Thermosphaera aggregans strain 3507 chromosome, Reference Sequence: NZ_CP063144.1: 483817-483892 tRNA-Gly

GCGGCGGTCGTCTAGCC**TGG**ACTAGGACGCCGGC**CTGCCA**CGCCGGAAATCCCGGG**TTCAA**ATCCCGGCCGCCGCA

Desulfurococcus mucosus DSM 2162, complete sequence NCBI Reference Sequence: NC_014961.1: c1034649-1034574 tRNA-Gly

GCGGCGGTCGTCTAGCC**TGG**ACTAGGACGCCGGC**CTGCCA**CGCCGGAAATCCCGGG**TTCAA**ATCCCGGCCGCCGCA

Desulfurococcus fermentans DSM 16532, complete genome GenBank: CP003321.1: 47280-47355 tRNA-Gly

GCGGCGGTCGTCTAGCC**TGG**ACTAGGACGCCGGC**CTGCCA**CGCCGGAAATCCCGGG**TTCAA**ATCCCGGCCGCCGCA

Caldiarchaeum subterraneum DNA, complete genome, from subsurface mine microbial mat GenBank: BA000048.1: c1207947-1207874 tRNA-Gly

GCGGCGGTCGTCTAGCC**TGGTA**GGACACCAGC**CTGCCA**CGCTGGGAGTCGCGGG**TTCAA**ATCCCGCCCGCCGCA

Cenarchaeum symbiosum A, complete genome GenBank: DP000238.1: c1160652-1160579 tRNA-Gly

GCAGTCATAGTATAGTT**TGGT**TATTATTCCAGCT**TGCCA**AGTTGGTGACCCGGG**T**C**CAA**ATCCCGGTGACTGCA

Nitrosopumilus maritimus SCM1, complete genome GenBank: CP000866.1: c397959-397883 tRNA-Gly

GCAGTTATAGTACAGTC**TGGT**TAGTACTCGTGCT**TGCCA**AGTACGTGACCCGGG**TTCAA**ATCCCGGTAACTGCACCA

Pyrococcus furiosus DSM 3638, complete genome GenBank: AE009950.1: c361464-361387 tRNA-Gly

GCGGTGGTAGTCTAGCC**TGGT**CTAGGACGCCACC**CTGCCA**AGGTGGAGACCCGGG**TTCAA**ATCCCGGCCACCGCACCA

Pyrococcus sp. NA2, complete sequence NCBI Reference Sequence: NC_015474.1: 1071591-1071668 tRNA-Gly

GCGGTGGTAGTCTAGCC**TGG**CCTAGGACGCCACC**CTGCCA**AGGTGGAGACCCGGG**TTCAA**ATCCCGGCCACCGCACC

Pyrococcus abyssi GE5, complete sequence NCBI Reference Sequence: NC_000868.1: c1468795-1468718 tRNA-Gly

GCGGTGGTAGTCTAGCC**TGG**CCTAGGACGCCACC**CTGCCA**AGGTGGAGACCCGGG**TTCAA**ATCCCGGCCACCGCACCA

Thermococcus sibiricus MM 739 tRNA-Gly-GCC-1-1 gene GenBank: HG986370.1

GCGGTGGTAGTCTAGCC**TGGT**CTAGGACAGCGGC**CTGCCA**CGCCGCGGGCCCGGG**TTCAA**ATCCCGGCCA
CCGCACCA

Thermococcus kodakarensis KOD1 DNA, complete genome GenBank: AP006878.1: c1311151-1311075 tRNA-Gly

GCGGTGGTAGTCTAGCC**TGG**CTAGGACAGCGGC**CTGCCA**CGCCGCTGGCCCGGG**TTCAA**ATCCCGGCCAC
CGCACCA

Thermococcus gammatolerans EJ3 tRNA-Gly-GCC-1-1 gene GenBank: HG986232.1

GCGGTGGTAGTCTAGCC**TGG**CTAGGACAGCGGC**CTGCCA**CGCCGCTGGCCCGGG**TTCAA**ATCCCGGCCACCGCACCA

Methanopyrus kandleri AV19, complete genome GenBank: AE009439.1: c1634571-1634496 tRNA-Gly

GCGGCCGCAGTCTAGTC**TGGTA**GGACGCGGGC**CTGCC**GAGCCCGTGGCCCGGG**TTCAA**ATCCCGGCGGCCGCACCA

Methanothermobacter thermautotrophicus strain WHS NODE_1_length_560865_cov_91_9211, whole genome shotgun sequence NCBI Reference Sequence: NZ_QKOF01000006.1: c518685-518612 tRNA-Gly

GCGGCGTTAGTCCAGCC**TGGT**TAAGACACTGGC**CTGCCA**CGCCAGCGACCCGGG**TTCAA**ATCCCGGACGCCGCA

Methanobrevibacter smithii strain WWM1085 Contig_1, whole genome shotgun sequence NCBI Reference Sequence: NZ_NQLD01000012.1: c378964-378891 tRNA-Gly

GCGGTGTTAGTCCAGCC**TGGT**TAAGACTCTAGC**CTGCCA**CGTTAGAGACCCGGG**TTCAA**ATCCCGGACGCCGCA

Methanocaldococcus bathoardescens strain JH146 chromosome, complete genome NCBI Reference Sequence: NZ_CP009149.1: c640173-640098 tRNA-Gly

GCGGCCTTGG**TG**TAGCC**TGGTA**ACACACGGGC**CTGCCA**CGCCCGGACCCCGGG**TTCAA**ATCCCGGAGGCCGCACCA

Methanocaldococcus infernus ME, complete genome GenBank: CP002009.1: c1047961-1047886 tRNA-Gly

GCGGCCTTGG**TG**TAGCC**TGGTA**ACACACGGGC**CTGCCA**CGCCCGGACCCCGGG**TTCAA**ATCCCGGAGGCCGCACCA

Methanocaldococcus fervens AG86, complete genome GenBank: CP001696.1:c280371-280296 tRNA-Gly

GCGGCCTTGG**TG**TAGCC**TGGTA**ACACACGGGC**CTGCCA**CGCCCGGACCCCGGG**TTCAA**ATCCCGGAGGCCGCACCA

Methanocaldococcus jannaschii DSM 2661, complete sequence NCBI Reference Sequence: NC_000909.1: 1150145-1150220 tRNA-Gly

GCGGCCT**TG**GTGTAGCC**TGGTA**ACACACGGGC**CTGCCA**CGCCCGGACCCCGGG**TTCAA**ATCCCGGAGGCCGCACCA

Methanococcus aeolicus Nankai-3, complete genome GenBank: CP000743.1: c846618-846546 tRNA-Gly

GCGGTTTT**GATG**TAGCC**TGGTA**TCATACGGCC**CTGCCA**CGGCCGACACCCGGG**TTC**G**AA**TCCCGGAGACCGCA

Methanococcus voltae strain DSM 14649 Ga0466840_02, NCBI Reference Sequence: NZ_JAGGMO010000002.1: c253803-253733 tRNA-Gly

GCGGCCTTG**ATG**T**A**G**TGGTA**TCATACGGCC**CTGCCA**CGGCCGATACCCGGG**TTC**G**AA**TCCCGGAGGCCGCA

Methanococcus vannielii SB gene GenBank: HG986108.1 tRNA-Gly

GCGGCTTT**GATG**TAG**A**C**TGGTA**TCATACGGCC**CTGCCA**CGGCCGACACCCGGG**TTCAA**ATCCCGGAGGCCGCA

Methanococcus maripaludis strain S2, complete sequence GenBank: BX950229.1: c1269120-1269048 tRNA-Gly

GCGGCTTTG**ATG**TAG**A**C**TGGTA**TCATACGGCC**CTGCCA**CGGCCGACACCCGGG**TTCAA**ATCCCGGAGGCCGCA

Aciduliprofundum boonei T469, complete genome GenBank: CP001941.1: c934319-934247 tRNA-Gly

GCGGGTG**TG**GTGTAGCC**TGGTA**ACACGAGGCC**CTGCCA**CGGCTTTGCCCCGGG**TTCAA**ATCCCGGCACCCGCA

Thermoplasma volcanium GSS1, complete sequence NCBI Reference Sequence: NC_002689.2: 551951-552023 tRNA-Gly

GCGGGTGTGGTGTAGCC**TGG**CAACACGCGAGCT**TGCCA**AGCTCGTGCCTCGGG**TTCAA**ATCCCGACATCCGCA

Ferroglobus placidus DSM 10642, complete genome GenBank: CP001899.1: 566151-566224 tRNA-Gly

GCGCCGGTGGTGTAGCC**TGG**CTAACACAGGGGCT**TGCC**GAGCCCCTGCCCCGGG**TTCAA**ATCCCGGCCGGCGCA

Archaeoglobus profundus DSM 5631, complete sequence NCBI Reference Sequence: NC_013741.1: 1365143-1365216 tRNA-Gly

GCGCCGGTGGTGTAGCC**TGG**CTAACACAGGGGC**CTGCCA**CGCCCCAGCCCCGGG**TTCAA**ATCCCGGCCGGCGCA

Archaeoglobus fulgidus isolate Archaeoglobus_fulgidus_oil, whole genome shotgun sequence NCBI Reference Sequence: NZ_FJNF01000147.1: 2080-2152 tRNA-Gly

GCGCCG**ATG**GTGTAGCC**TGGTA**ACACAGGGGCT**TGCC**GAGCCCCTGCCCCGGG**TTCAA**ATCCCGGTCGGCGCA

Methanocella paludicola SANAE, complete sequence NCBI Reference Sequence: NC_013665.1: 2598124-2598195 tRNA-Gly

GCGCCGGTAGTGT**A**G**TGGT**TATCACTATGGCT**TGCCA**AGCCATAAACTCGGG**TTC**G**A**GTCCCGACCGGCGCA

Methanosaeta harundinacea 6Ac, complete sequence NCBI Reference Sequence: NC_017527.1: c235976-235905 tRNA-Gly

GCGCCGATAGTGT**A**G**TGGT**CATCACTAGGCGT**TGCCA**ACGCCTAAACCCGGG**TTC**G**A**ATCCCGGTCGGCGCA

Methanosarcina barkeri CM1, complete genome GenBank: CP008746.1: 1891060-1891131 tRNA-Gly

ACATCAGTAGTGT**A**GC**GGT**CATCACCGGGCGT**TGCCA**ACGCTCGAACCCGGG**TTC**G**A**ATCCCGGCTGATGTA

Methanosarcina mazei TMA DNA, complete genome GenBank: AP019780.1: 633659-633730 tRNA-Gly

ACATCAGTAGTGTAGC**GGT**CATCACCGGGCGT**TGCCA**ACGCTCGAACCCGGG**TTC**G**A**ATCCCGGCTGGTGTA

Methanococcoides burtonii DSM 6242, complete genome GenBank: CP000300.1: c113010-112939 tRNA-Gly

ACACCAGTAGTGTAGC**GGT**TATCACCGGGCGT**TGCCA**ACGCTCGAACTCGGG**TTC**G**A**TTCCCGACTGGTGTA

Methanocorpusculum labreanum Z, complete sequence NCBI Reference Sequence: NC_008942.1: 337952-338023 tRNA-Gly

GCATCGATAGTGT**A**G**TGGT**TATCACTAGGCGT**TGCCA**ACGCCTAAACTCGGG**TTC**G**A**GTCCCGGTCGATGCA

Methanospirillum hungatei strain GP1 chromosome, Reference Sequence: NZ_CP077107.1: 1403216-1403287 tRNA-Gly

GCGTTGATAGTGTAG**TGGT**TATCACTAGGCGT**TGCCA**ACGCCTAAACCCGGG**TTC**G**A**GTCCCGGTCAGCGCA

Methanoregula boonei 6A8, complete sequence NCBI Reference Sequence: NC_009712.1: 1259143-1259214 tRNA-Gly

GCGCCGATAGTGT**A**G**TGGT**TATCACTAGGCGT**TGCCA**ACGCCTAAACTCGGG**TTC**G**A**GTCCCGATCGGCGCA

Natrialba magadii ATCC 43099, complete sequence NCBI Reference Sequence: NC_013922.1: c2067059-2067024, c2067011-2066964 tRNA-Gly

GGAAGGGTCGCTCAGC**GGTA**GAGCATCGCGG**TGCCA**CCGCGACGGCAGCACATGCCTCGTGGCG**TTCAA**A
TCCCACCCCTTCCG

Halobacterium salinarum strain 91-R6 chromosome, complete genome GenBank: CP038631.1: c1621908-1621851 tRNA-Gly

CCGACGTCGAATAG**A**G**TGGTA**TCACGTGACCT**TGCCA**TGGTCACAACCTGGG**TTCAA**ATCCCAGCCAGCGCA

Haloferax volcanii strain H98 scaffold_2, whole genome NCBI Reference Sequence: NZ_JAERQU010000003.1: 84353-423 tRNA-Gly

GCGCTGGTAGTGT**A**G**TGGTA**TCACGTGACCT**TGCCA**TGGTCACAACCTGGG**TTCAA**ATCCCAGCCAGCGCA

Natronomonas pharaonis DSM 2160 complete genome GenBank: CR936257.1: c2368761-2368691 tRNA-Gly

GCGCTGGTAGTGT**A**G**TGGTA**TCACGTGACCT**TGCCA**TGGTCACAACCCGAG**TTCAA**ATCTCGGCCAGCGCA

Halomicrobium mukohataei strain pws1 Scaffold_1, whole genome shotgun sequence NCBI Reference Sequence: NZ_WOYG01000001.1: c1749471-1749401 tRNA-Gly

GCGGTGGTAGTGT**A**G**TGGTA**TCACAGGACC**CTGCCA**CGGTCCTAACGGGGG**TTCAA**ATCCCCCCCACCGCA

Haloarcula marismortui ATCC 43049 chromosome, NCBI Reference Sequence: NZ_CP039138.1: 1034685-1034756 tRNA-Gly

GCGCTGGTAGTGTAGT**TGGTA**TCACGTGACCT**TGCCA**TGGTCACAACCTGGG**TTCAA**ATCCCAGCCAGCGCA

Haladaptatus paucihalophilus DX253 B208DRAFT_contig1.1_C, whole genome shotgun sequence NCBI Reference Sequence: NZ_AQXI01000001.1: 1281796-1281866 tRNA-Gly

GCGCTGGTAGTGT**A**G**TGGTA**TCACGTGACCT**TGCCA**TGGTCACAACCTGGG**TTCAA**ATCCCAGCCAGCGCA

Natrinema pellirubrum DSM 15624, complete genome GenBank: CP003372.1: c854337-854267 tRNA-Gly

GCGCTGGTAGTGT**A**G**TGGTA**TCACGTGACCT**TGCCA**TGGTCACAACCTGGG**TTCAA**ATCCCAGCCAGCGCA

Natronobacterium gregoryi SP2, complete sequence NCBI Reference Sequence: NC_019792.1: 3540910-3540980 tRNA-Gly

GCGCTGGTAGTGT**A**G**TGGTA**TCACGTGACCT**TGCCA**TGGTCACAACCTGGG**TTCAA**ATCCCAGCCAGCGCA

Phocaeicola abscessus CCUG 55929, whole genome shotgun sequence NCBI Reference: NZ_HE997184.1: c142655-142583 tRNA-Gly

GCGGAAATAGCTCAGT**TGGTA**GAGCACGACCT**TGCCA**AGGTCGGGGTCGCGAG**TTC**G**A**GTCTCGTTTTCCGCT

Bacteroides massiliensis B84634 = Timone 84634 = DSM 17679 = JCM 13223 genomic scaffold aczJl-supercont1.1, whole genome shotgun sequence GenBank: KB905472.1: c1648521-1648446 tRNA-Gly

GCGGAAATAGCTCAGT**TGGTA**GAGCATAACCT**TGCCA**AGGTTAGGGTCGCGAG**TTC**G**A**GTCTCGTTTTCCGCTCTA

Bacteroides heparinolyticus strain F0111 chromosome, complete genome NCBI Reference Sequence: NZ_CP027234.1: 599826-599898 tRNA-Gly

GCGGAAATAGCTCAGT**TGGTA**GAGCACAACCT**TGCCA**AGGTTGGGGTCGCGAG**TTC**G**A**GTCTCGTTTTCCGCT

Bacteroides fragilis NCTC 9343, complete genome GenBank: CR626927.1: 537974-538046 tRNA-Gly

GCGGAAATAGCTCAGT**TGGTA**GAGCATAACCT**TGCCA**AGGTTAGGGTCGCGAG**TTC**G**A**GTCTCGTTTTCCGCT

Bacteroides finegoldii strain D53t1_180928_A7 NODE_3_length_206098_cov_23.2893, whole genome shotgun sequence NCBI Reference Sequence: NZ_JADNLJ010000003.1: 45624-45696 tRNA-Gly

GCGGAAATAGCTCAGT**TGGTA**GAGCATAACCT**TGCCA**AGGTTAGGGTCGCGAG**TTC**G**A**GTCTCGTTTTCCGCT

Bacteroides thetaiotaomicron strain 7330 chromosome, NCBI Reference Sequence: NZ_CP012937.1: 2784339-2784411 tRNA-Gly

GCGGAA**AT**AGCTCAGT**TGGTA**GAGCATAACCT**TGCCA**AGGTTAGGGTCGCGAG**TTC**G**A**GTCTCGTTTTCCGCT

Porphyromonas asaccharolytica DSM 20707, complete sequence NCBI Reference Seq: NC_015501.1: 369164-369236 tRNA-Gly

GCGGAAGTAGCTCAGT**TGGTA**GAGCACAACCT**TGCCA**AGGTTGGGGTCGCGGG**TTC**G**A**GTCCCGTCTTCCGCT

Table S2. tRNAs from various species; Methanopyrus kandleri other than tRNA-Gly^{GCC}, bacteria, bacteroides and mammals

Methanopyrus kandleri AV19, complete sequence NCBI Reference Sequence: NC_003551.1: c37227-37152 tRNA-Gly^{TCC}
GCGGCCGCAGTCTAGTC**TGGTA**GGACGCGGGC**CTTCC**GAGCCCGTGGCCCGGG**TTCAA**ATCCCGGCGGCCGCACCA
Methanopyrus kandleri AV19, complete sequence NCBI Reference Sequence: NC_003551.1: 51546-51622 tRNA-Ala^{GGC}
GGGCCCGCAGCTCAGCC**TGG**CAGAGCGCGGCCT**TGGC**GCGGCCGTGGCCCCGGG**TTCAA**ATCCCGGCGGGTCCACCA
Methanopyrus kandleri AV19, complete sequence NCBI Reference Sequence: NC_003551.1: 51735-51812 tRNA-Ile^{GAT}
GGGCCCGCAGCTCAGCCA**GGT**TAGAGCGCGGGG**CTGATA**ACCCCGAGGTCCCGGG**TTCAA**ATCCCGGCGGGCCCACCA
Methanopyrus kandleri AV19, complete sequence NCBI Reference Sequence: NC_003551.1: 384999-385073 tRNA-Ile^{CAT}
GGGCCCGCAGCTCAGTCC**GGT**TAGAGCGCGGGG**CTCATA**ACCCCGTGGTCCCGGG**TTCAA**ATCCCGGCGGGCCCA
Methanopyrus kandleri AV19, complete sequence NCBI Reference Sequence: NC_003551.1: 55068-55107,55184-55221 tRNA-Trp^{CCA}
GGGCCCGCGGTGTAGCCA**GGT**CTATCATGCGGGG**CTCCA**GACCCCGTGACCCGGG**TTCAA**ATCCCGGCGGGCCCACCA
Methanopyrus kandleri AV19, complete sequence NCBI Reference Sequence: NC_003551.1: c93290-93212 tRNA-Met^{CAT}
AGCGGGGCGG**GGTA**GCCAGGTCCATCCCGCCGGG**CTCATA**ACCCGGAGGTCCCGGG**TTCAA**ATCCCGGCCCCGCTACCA
Methanopyrus kandleri AV19, complete sequence NCBI Reference Sequence: NC_003551.1: 172302-172377 tRNA-GLN^{TTG}
AGGGCCGTGG**GGTA**GCGGTCTATCCTGCGGGG**CTTTG**GACCCCGCGACCCCGG**TTCAA**ATCCGGGCGGCCCTACCA
Methanopyrus kandleri AV19, complete sequence NCBI Reference Sequence: NC_003551.1: 188239-188327 tRNA-Sec^{TCA}
GGCCGCCGCCACCGGGG**TGGT**CCCCGGGCCGGA**CTTCA**GATCCGGCGCGCCCCGAGTGGGGCGCGGGG**TTCAA**TTCCCCGCCA
Methanopyrus kandleri AV19, complete sequence NCBI Reference Sequence: NC_003551.1: c272692-272615 tRNA-Thr^{TGT}
GCCGCGGCAGCTTAGCC**TGGT**TAGAGCGCGGGA**CTTGTA**ATCCCGTGGTCCCGGG**TTCAA**ATCCCGGCCGCGGCTCCA
Methanopyrus kandleri AV19, complete sequence NCBI Reference Sequence: NC_003551.1: 563671-563760 tRNA-Leu^{TAA}
GCGGGGGCGCCCGAGCC**TGGT**CGAAGGGGGCGGA**CTTAA**GATCCGCTGGCCGTAGGGCCGTCCCGGG**TTCAA**ATCCCGGCCA
Methanopyrus kandleri AV19, complete sequence NCBI Reference Sequence: NC_003551.1: c512322-512249 tRNA-Arg^{GCG}
GGCCCCGCAGTCTAGCC**TGG**ATAGGACGCGGGC**CTGCG**GAGCCCGTAGCCCGGG**TTCAA**ATCCCGGCGGGGCCA

>Bacillus_anthracis_Ames_chr.trna50:GlyGCC<(746745:746819)<<Gly<(GCC)
GCGGAAGTAGTTC**A**G**TGGTA**GAATACAACCT**TGCCA**AGGTTGGGGTCGCGGG**TTC**G**A**ATCCCGTCTTCCGCTCCA
>Bacillus_anthracis_Ames_chr.trna45:GlyTCC<(543071:543144)<<Gly<(TCC)
GCGGG**TG**TAGTTT**A**G**TGGTA**AAACAAGAGC**CTTCCA**AGCTCTGGTCGAGAG**TTC**G**A**TTCTCTTCACCCGCTCCA
>Bacillus_cereus_ATCC_10987_chr.trna15:GlyGCC<(150984:151055)<<Gly<(GCC)
GCGGAAGTAGTTC**A**G**TGGTA**GAATACAACCT**TGCCA**AGGTTGGGGTCGCGGG**TTC**G**A**ATCCCGTCTTCCGCTCCA
>Bacillus_cereus_ATCC_10987_chr.trna27:GlyTCC<(279452:279522)<<Gly<(TCC)
GCGGG**TG**TAGTTT**A**G**TGGTA**AAACAAGAGC**CTTCCA**AGCTCTGGTCGAGAG**TTC**G**A**TTCTCTTCACCCGCTCCA
>Bacillus_licheniformis_ATCC_14580_chr.trna55:GlyGCC<(3116926:3116855)<<Gly<(GCC)
GCGGAAGTAGTTC**A**G**TGGTA**GAACACCACCT**TGCCA**AGGTGGGGGTCGCGGG**TTC**G**A**ATCCCGTCTTCCGCTCCA
>Bacillus_licheniformis_ATCC_14580_chr.trna23:GlyTCC<(611775:611848)<<Gly<(TCC)
GCGGG**TG**TAGTTT**A**G**TGGTA**AAACCTCAGC**CTTCCA**AGCTGATGTCGTGAG**TTC**G**A**TTCTCATCACCCGCTCCA
>Bacillus_thuringiensis_Al_Hakam_chr.trna84:GlyGCC<(4667815:4667741)<<Gly<(GCC)
GCGGAAGTAGTTC**A**G**TGGTA**GAATACAACCT**TGCCA**AGGTTGGGGTCGCGGG**TTC**G**A**ATCCCGTCTTCCGCTCCA
>Bacillus_thuringiensis_Al_Hakam_chr.trna36:GlyTCC<(258057:258127)<<Gly<(TCC)
GCGGG**TG**TAGTTT**A**G**TGGTA**AAACAAGAGC**CTTCCA**AGCTCTGGTCGAGAG**TTC**G**A**TTCTCTTCACCCGCTCCA
>Bacillus_weihenstephanensis_KBAB4_chr.trna24:GlyGCC<(156592:156663)<<Gly<(GCC)
GCGGAAGTAGTTC**A**G**TGGTA**GAATACAACCT**TGCCA**AGGTTGGGGTCGCGGG**TTC**G**A**ATCCCGTCTTCCGCTCCA
>Bacillus_weihenstephanensis_KBAB4_chr.trna37:GlyTCC<(247654:247724)<<Gly<(TCC)
GCGGG**TG**TAGTTT**A**G**TGGTA**AAACAAGAGC**CTTCCA**AGCTCTGGTCGAGAG**TTC**G**A**TTCTTTCACCCGCTCCA

>Bacteroides_fragilis_YCH46_chr.trna52:GlyGCC<(2904501:2904426)<<Gly<(GCC)
GCGGAAATAGCTCAGT**TGGTA**GAGCATAACCT**TGCCA**AGGTTAGGGTCGCGAG**TTC**G**AG**TCTCGTTTTCCGCTCAA
>Bacteroides_fragilis_YCH46_chr.trna55:GlyCCC<(2858492:2858417)<<Gly<(CCC)
GCGGTAGTAGCTCAGT**TGG**C**A**GAGCGGCGGCT**TCCCA**AGCCGCAGGTCACGAG**TTC**G**A**CCCTCGCCTACCGCTCTA
>Bacteroides_vulgatus_ATCC_8482_chr.trna69:GlyGCC<(1160616:1160544)<<Gly<(GCC)
GCGGAAATAGCTCAGT**TGGTA**GAGCATAACCT**TGCCA**AGGTTAGGGTCGCGAG**TTC**G**AG**TCTCGTTTTCCGCT
>Bacteroides_vulgatus_ATCC_8482_chr.trna29:GlyCCC<(3943164:3943236)<<Gly<(CCC)
GCGGCAGTAGCTCAGT**TGGTA**GAGCATCAGCT**TCCCA**AGCTGAGGGTCACGAG**TTC**G**A**ACCTCGCTTGCCGCT

>Bos_taurus_chr3.trna2984:GlyGCC<(86322865:86322938)<<Gly<(GCC)
TCCCTGG**TG**GTCT**A**G**TGGT**TTAAGACTCTGCA**CTGCCA**ATACAGCAGGCCCAGG**TTCAA**TCCCTGGTCAGGGAG
>Bos_taurus_chr3.trna2384:GlyTCC<(68084172:68084244)<<Gly<(TCC)
TCCCTGGTAGTCT**A**G**TGGT**TAAGACTCTGTG**CTTCCA**CTGCAGGGGGCTCAGG**TTCAA**ACTCTGGTTGGGGAA
>Homo_sapiens_chr21.trna2:GlyGCC<(18827177:18827107)<<Gly<(GCC)
GCATGGG**TG**GTTC**A**G**TGGTA**GAATTCTCGC**CTGCCA**CGCGGGAGGCCCGGG**TTC**G**A**TTCCCGGCCCATGCA
>Homo_sapiens_chr1.trna4:GlyCCC<(17188416:17188486)<<Gly<(CCC)
GCATTGG**TG**GTTC**A**G**TGGTA**GAATTCTCGC**CTCCCA**CGCGGGAGACCCGGG**TTCAA**TTCCCGGCCAATGCA

Table S3. Percentage of AL-pentamers in rRNAs or mRNAs of proteins participating to the ribosomal translation in Methanococcus voltae

Methanococcus voltae DNA polymerase gene, complete cds GenBank: L33366.1

TATGGATTTAGATTATAATTCCAAAGACCTTTGTATTGATATGTATTACAAAAATTGTGGACTAAAAAAA
CCCGAAATAAATTTACAAAAA**GAATG**TGAGTTTAAACCTTATTTCTATGTAGATACATCAGAACCAAAAG
AAATTTACGATTATTT**AGATG**GCCTAAA**TCAAGA**AATTGATTTAAAAAAATTAGAACC**TGAAT**TTGAAAA
TAATACATCCTTAAAAGTTCAGGATTTAATAACAAACATAGAAATTATTGAAAAAATTGTTTATTCTGAT
TATATTT**TGAATGG**AAAGGATATTTCAGAAGTAAGTGATTTTAAAAATAAAAAAGAACGAAAAATATGTA
AAGTCTATGTAAAATATCCTAATCACGTGAAAATAATAAGAGAATACTTTAAAGAATTTGGAAAGTCCT**A**
TGAATTTGA**CATTC**CTTTTTTAAGGAGATATATGATTGAC**CAAGAT**ATTGTACCATCGGCAAAATATTCC
G**AAGAT**AAT**AAGAT**AGACAACAGTATTCCAGAATTAAATTGTATCGCCTTTGATATGGAACTATATTGTA
AAAAAGAACCAAATGCAAAGAAAGACCCTATTAT**AATGGT**TAATTTATTCTCTAAAGACTACCAAAAAGT
AATTACCTATAAAAAATTTGAAAATTCGGAATAT**AATGG**ATGTGTGGATTACGTAA**AAGATGAA**AAAGAA
TTG**ATTCAA**AAAACAATTGAAATTTTGAAACAATATGACGTAATATATACGTAT**AATGG**AGATAATTTTG
ATTTCCCATATTTAAAGAAAAGAGCAAACATTT**ATGAA**AT**TGAAT**TAGATTTTGACAATGCTTCGAATTC
TCAACAACCTCAAATCATAAAAAT**TTCAA**AAGGGGGAATCAATAGAAAGAGTAAAATTCCAGGAATTATC
CACATTGACCTCTACCCAATAGCTAGAAAATTATTAAATCTTACCAAATATAAATTAGA**GAATG**TAGTAC
AGGAATTATTTAAAATAAACAAAGAAGCTGTCGATTATGGCGATATTCCAAAAATGTGGGAAACCG**AAGA**
TACTACATTGCTGAGATATGCCT**ATGAAGATG**CACTATATACCTATAA**AATGG**GGAATTACTTCTTACCA
CTTGAAATAATGTTCTCTAGAATAGTTAATCAGCCCTTGTATGATACTAGTA**GAATGAAT**AGTAGCCA**AA**
TGGTGGAATTTTTATTACTTAAACGGTCTTTTGAACAAAATATGATTTCTCCAAACCGTCC**TTCAAGTTC**
AAGTTACAGAGAACG**TGCCA**AATTCTCTTACGAAGGTGGTTATGTTAGAGAACCACTAAAAGGT**ATTCAA**
GAAGATATCGTTTCACTTGACTTTATGAGTCTATATCC**TTCAA**TATTGATAA**GCCAT**AATATAAGCCCTG
AAACGGTCATTTACGAGGAAAAAGAACGAGAAAATATGGAGCTTGGGATAATTCCTAAAACCCTTA**ATGA**
ATTATTAAGTCGAAGAAAACATATTAAAATGCTATTAA**AAGAT**AAA**ATTCAA**AAAAATGAGTTT**GATGAA**
GAATATAGTAGATTAGAACATGAGCAAAAAT**CCATT**AAAGTGCTTGCAAATA**GCCATTATGGT**TATTTAG
CATTCCCGATGGCTCGC**TGGTACT**CGGATAAATGTGCGGA**AATGGT**TACAGGACT**TGGTA**GAAAATAT**AT**
TCAAGAAA**CCATT**GAAAAAGCAGAAGAATTTGGATTTAAGGTAATTTATGCAGATACCG**ATGGT**TTTTAT
GCAA**AATGG**GATTATGATAAAC**TTCAA**AAAGGTAAAAAAGAAGAAAATGATAAATCTGATAAATTATCTA
ATTTACCTAAATTATCAAAAGAAGAATTGATAATATTAACTAAAAAGTTCTTAAAAGGAATTA**ATGAA**GA
ATTACCAGAAGGTATGGAATTAGAATTTGAAGGACATTTTAAAAGAGGGTTATTCGTTACAAAAAAGAAA
TATGCGTTAATCG**AAGATGA**CGGGCATATTGTAGTAAAAGGTTTGGAAGTTGTAAGACGAGATTGGTCAA
ACATTGCTA**AAGAT**ACTCAACAAGCCGTTATTAGAGCACTATTGG**AAGATGGT**GACGTTAATTTAGCTAA
AAAAATTATTAAAAATACGATTGATAATTTGAAAAAAGGAAATATTGATAAAAATGATTTGCTTATACAT
ACTCAATTAACTAAAAATATTGAGGAATATAAGTCA**ACTGC**ACCACATATTGAAGTAGCTAAAAAAATAA
AGCAGCGTGGTG**ATTCA**GTCCGAGTAGGGGATGTTATAAGTTATATAATTGTCAAAGGTAGCCG**TTCAA**T
TAGTGAAAGAGCTGAACTATTAGAGTATGCTGGCGATTATGATATTAATTACTACATTGACAATCAGGTT
TTACCACCCGTTATTAGGATTATGGAGTCTTTGGGAATTTCGG**AAGATGAAT**TGAAAA**ATTCA**GGTAAGC
AGTTTAAACTCGACCAATTTATGTAA

92 2472 **observed: 3.7% expected: 2.1% ± 0.5 (95% confidence interval)**

Methanococcus voltae strain DSM 14649 Ga0466840_02, whole genome shotgun sequence NCBI Reference Sequence: NZ_JAGGMO010000002.1: c172863-172297 DNA-directed RNA polymerase subunit D

ATGCAAAAAGAGGTAAAAAGAACCGGGGAAATTATAACT**ATGAA**AGTTGAAGCTCCTTTGTCTTTTTCCA
GTGCATTAAGAAGAATAATGATATCAGAATTACCAACTTATGCTATAGAAAATGTTTTTTTCT**ATGAA**AA
CACATCATCAATGTAC**GATGAA**GTTTTAGCTCATAGGTTAGGT**ATGGTAC**CTATAAAAGGAAAACCTGTA
AGTTCT**GATGAAT**TAATAACATTTGTAGTATCTAAAGAAGGACCTTGCACAGTTTACTCATCAGATTTAA
AATCAGAAGTTGGGGAACCTGCTTTTGAGAACATACCTTTGGTTAAATTGTCAGAAGGTCAAAAATTAGA
AGC**TGAATG**TGAAGCAT**TGGTA**GGAACCGGTAAA**ATTCATTCAAAATGG**CAACCATGCAACG**TGGTA**TAT
ACACAACTCG**AAGAT**AACCTCGTAGAGTTT**AAGAT**AGAATCACACAAAAATATGGAAGCTGAGGATTTAG
T**AAGAT**CTGCATTAGAAATTCTTAAAAATAAGGCAGAAACCTGCTTAATT**TGAAT**TAGAAAGTCATGTATT
TAATTAA

22 563 **observed: 3.9% expected: 2.1% ± 1 (95% confidence interval)**

Methanococcus voltae A3, complete genome GenBank: CP002057.1: 1754095-1755849 Glycyl-tRNA synthetase

ATGGAAAAAATCGATAAAAATGAAAAATATAATAAAATAATGGATTTAATCAAAAGAAGAGGTTATTTGT
GGAATTCCTTTGAAATTTATGGGGGAATCGCAGGTTTCTTTGATTATGGGCCATTAGGTGCTATTTTAAA
AAATAACATCGTAAATACGTGGAGAAAACATTATATTATTAATGAAGGATTTTACGAAATAGAGGGACCT
ACAATAAATCCTTATGAAGTTTTAAAAGCTTCTGGACACGTAGACAACTTTACGGACCCATTAGTACAAT
GTAAAGAATGTAATGAGTCATTTAGGGCAGACCACATAATTGAAGAAAATGTTGATATTGATACCGAAGG
TAAAACACTCGATGAATTACAAGCTTTAATCACTGAAAACGATATAAAATGTCCACTTTGTGGCGGTGAA
TTTAAAGAAGTTGACACTTTCAATCTAATGTTTAAAACTTCAATCGGACCAAGCGGTAAAAGAGTAGCAT
TTATGAGACCGGAAACCGCACAAGGTATATTTATCCAATTTAAAAGAATAAACCAGTTTTTTAGAAATAA
ATTACCGTTCGGGGCTGTTCAAATAGGTAAATCATACAGGAATGAAATTTCACCAAGACAGGGAGTAATA
AGACTTAGGGAATTTAGCCAAGCTGAAGCTGAATTTTTCATACACCCTGATAAAAAGAACTATGAGAAGT
TTGAACGTGTAAAAGATATGGTTTTACCATTATTGCCTTCAAATAACCAAGAAGACGAAACATTGTCTGC
AGAGGATAAAATCGTTAAAATGACTTTGGGTGAAGCTGTAGAAAAAGGTATCGTAAAAAATAAAGCTATT
GCATACTTTATTTCAATAACTGCTAATTTCTTACTCGACGTCGGAATAGATGCAAGTAAAATGAGATTTA
GACAACACTTGCCTAACGAAATGGCACACTATGCTTCAGATTGTTGGGATGCAGAACTTTATTCCGATAG
ATTTGGATGGGTTGAATGTGTGGGTGTAGCAGATAGAACCGATTACGATTTAAAATCACATATGGCTACA
AGTGGTGAAGATTTAAGCGTTTTCGTAGAATATGAAGAACCTAAGGAAATTGAAACCTTTGATATTGAAT
TAAACTTTAAAGCACTGGGAAAAACATTTAAAGGAGATTCAAAAATAATTCAAAAATTAATTTCAGAAAT
GGATTATCAAAAAATTGAAGAAATGGTTTCCGGCATCGAAAATAATGAAAAAGGCAATAAATATATTTTA
AAAGCAGAAGATGGTAAGGAATTTGAATTAACAAGTGAATACCTCACTGCTAAAAAGATAACTAAAACAG
TAAACGGCGAAAAAGTAATTCCTCACGTTATTGAGCCTTCATACGGTGTTGATAGGATTACTTACTTTGT
TATTGAACACGCTTTCAATGAAGAAGAAGACAGAACCTTTATGAAATTAGCACCAAGTGTAGCACCTATT
AAAGCGGGCGTTTTCCCATTAGTAAATAAAGAGCAAATGCCAATTATTGCAAGGGAAATCGAAGAAACAC
TTAGAAATTCGGGAATAATCGCAGAATATGACGATAGCGGTGCTATTGGTAGAAGATATTTAAGAATGGA
TGAGGTGGGTACACCGTTCTGCATCACCGTTGACGGTGAATCCTTAGATAGCGGAACCGTAACAATCCGT
GAAAGAGATGAAAGAACTCAAAAAAGATTAAAAATCGAAGAATTAGGTGAATATATTAATAATTCTTTAA
AATAA

62 1751 observed: 5.7% expected: 2.1% ± 0.6% (95% confidence interval)

Methanococcus voltae strain A 2 Ga0416753_02, whole genome shotgun sequence NCBI Reference Sequence: NZ_JAGGMU010000002.1: 191261-191872 ATPase subunit E

ATGGGAGCGGAAAAGATAACTTCCAAAATTAAAGAAGATGCAAAATTAAAAGCTGATTCAATCGTAGCTG
ACGCACAAGCTAAATACGAAGCAACAATGGCAGAAGCTAAAGAAGAAGCTGAAAAAAGAAAGCAAGCTAT
TCTTAGAAAAGGAGAAAAAGAAGCAGAAATGGCTGAAAACAGAATTTTAGCTGATGCAAGATTATCCTCA
AAAAAGAAATTGCTTGAAGAAAGAGAAAATACAATTCAAATGACACTCGAAAAATTAGAGGAAGACTTAA
TGAAATTACCTCAAAAAGATGAATACAAAGACATCTTAACAGGTATGGTAATTAAAGGCGTTCTCTCAAT
CGGTGGCGGAGAATTGACACTCGAAATGAACAAGAATGACTTCGAATTAATCGGTGACGAAACTTTGTGG
AAGCTCGAAAAAGAAGTCGAAGAAAAATTGAATATTGTAACAGTCTTGAAAAAAGGCGAACCAATCGATA
TAATCGGCGGATGTATTGTTAAATCAGCAGATGGTACAAAAGTATCCGACAACAGTCTTGAATCAACATT
CAAAAGAAATTTAGATAGTGTAAGAGCAAAAATAGCAGATTTATTATTCTAA

31 608 observed: 5.1% expected: 2.1% ± 1 (95% confidence interval)

Table S4. rRNAs and rproteins from different species presented in the evolutionary order by G. Caetano-Anollés (given in Harish, A., and G. Caetano-Anollés. 2012. Ribosomal history reveals origins of modern protein synthesis. PLoS ONE. 7:e32776) : Nb of observed pentamers among the pentamer set {ATTCA TTCAA TCAAG CAAGA AGATG GATGA ATGAA TGAAT} (in red), Nb of nucleotides in the sequence (in black), Nb of expected pentamers with its standard deviation (in red), Nb of standard deviations between observed and expected numbers (in blue).

Methanococcus maripaludis strain DSM 2067 chromosome, complete genome GenBank: CP026606.1: 1573556-1574872 S12

ATGGCAATTAAAAACGTTTCAATCGTGTACCCGAATAAATTTGGCGGAGGGATAGCATGTCTTGCAGTGC
ATGTTTTAACCGGCCACTTAAACAAGTACTCAGATATTTCTGCAAATGCGTATTTTATAGAAAATTACTC
GAAAATTAAGAACAATGACGCTATTTTAATAACTTTACAGTACGAAAACGACTATTTTAACGTTGTAAAA
ATTGTAAAAGAATTAAAAGCTCAAAATCCAAACGCTATTTTTATTGCAGGGGGTCCCTGCGCCATTTCAA
ACCCGCTAACTATTCAGGAATTTTTTGATGCATTTGTTGTCGGTGAAATTGAAGGAACTGATACAATGTA
CCAATTGATAAATGGTAATTTTGAAATTCCTGGAGTATATGTTCCGAAAAATTATAATAATGAAAAAATT
AAAAGAATATATCCAAAAAAACTCGGGATAGATGATTATCCAATAGCCCAAGTTACGCATGAATCGGGAG
CTTATGGAAAAGCTTATCTTCTTGAAATTGGAAGGGGTTGCATTAGAAACTGTAAGTTTTGCATGGCCAA

ATGCATCTACGCTCCTC**CAAGAT**ATCGAAAAATTG**AAGAT**TTAAAGTATCTGGTC**GATGAA**GGATTAAAA
AACACCGATGCAGACAAGGTTTCACTAATTGCCCCATCTGTAAGTGATTATAAGTAT**GTACT**AGATTTAT
GCGCACACATCTCTGAAAAAAATGTGTTAATTTCGCCTTCATCATTACGGGCAGATACAAT**TACTGATGA**
ACTGCTTGACTTTTTAAATCTGAAAACATTGACGATAGCACCCGAAGCAGGTAGCGAAGTTCTGCGAAAA
AAAATTGATAAAGGAGTCACTGAAGAAAATATTTTAAATGCAGTTGATATTGCTAAAACACACGGAATAT
CTACCGTAAAACTTTATTAT**ATGGT**TGGTTTTCCT**GATGAAT**CAG**AAGATGA**TATTGAAGAAATAATAAA
TTTAACAAAAAAAATAA**AAGAT**AACGTTCGAAAAGT**AGATG**TTAGCATAAACCC**AATGGT**TCCAAAGCCC
CACACTCCTTTCGAAGACTT**TGAAT**TTGACATGGACTCAAAAACG**AAGAT**AAAATATATCGAAAAAAGTC
TGCGAAAAATAAAAGTTAGTGTTGATTTTGAAAA**ATTCAA**TTCT**ATGGT**TGCACAGACCGTTCTTG**CAAG**
AGGGGGAAGTGAACT**TTCAA**AAGTTTTAAATGAG**TCAAGA**AACACAATCGAACTTATTAAAAATGTGGAT
TTGGATAATTACATTAATAAAATCGAAGAAAAACCTTGGGATTTTATAGAAATCTAA

27 1314 **10.26 (3.2) 5.2**

Methanococcus maripaludis strain DSM 2067 chromosome, complete genome GenBank: CP026606.1: 387848-388039 S17

TTGGGAAGAATAAGGCAAA**CATTCA**TCAAAAGAACCGGTGAAGAACTCATTGAGAAATTTGCAGACAAGT
TCACAAGCGATTTTGAAGAAAACAAAAAAGCTGTTGAAGAAGTTGCAATGATCTCAACAAAA**CCATT**GAG
AAACAGAATAGCAGGTTATGT**TACTGC**TAAAGTAAAAAAA**ATGAA**CGCATAA

2 188 **1.47 (1.2) 0.44**

Methanococcus maripaludis strain OS7mut1 chromosome, complete genome NCBI Reference Sequence: NZ_CP020120.1: 1380141-1380545 S9

GTGAAAGTAGTCCACACAGTAGGTAAAAGAAGAACCGCAATTG**CAAGA**GC**TACTGC**AAAAGAAGGAAGCG
GTAAAATCAGAATTAACAAAAAA**CCATT**AGAATT**AATGG**AACCTAAATACATCAAA**ATGAA**ATT**AATGG**A
ACCTGTTATCTTAGCAGGCGAAGCTTTAAGCAACATTGACGTAGATATTGATGTTAAAGGTGGCGGTATA
GTAAGCCA**AATGG**ATGCTA**CAAGA**ACCGCTTTAGGTAAAGCTATTGT**TGAAT**T**TACTGGTA**A**AATGG**ATT
TGAAAGAAAAATTCTTAAGTTACGACAGAACATTACTTGTCAGTGACG**CAAGA**AGAACCGAACCACACAA
ACCAAGTAAGTCCTCAAAAGGTC**CAAGA**GCTAAAAGACAGAAATCATACAGATAA

6 401 **3.13 (1.77) 1.62**

Methanococcus maripaludis strain DSM 2067 chromosome, complete genome GenBank: CP026606.1: c1304042-1303581 L22

ATGGCTAAACTAAAATACAAAGTTGAAGCTGACCCAAAGAAC**ACTGCAAGA**GCTATGGGCAGAACTTTAA
GAATA**TCAAGA**AAACATGCTATTGAACTTTGCAGAGAATTAAGTG**GAATG**AAATT**AGATG**CTGCTGTTGC
ATACTTAAACAGAGTAATCGCATTAGAAACTCCTGTT**CCATTCAA**AGTTCACAACAAAGACTTACCACAC
AGAAAAGGAAAAATTGGAACACACTCAGGTAGATTCCCACAGAAAGCTTCGTTAGAAATCTTAAACGTTT
TAGATAACGCTAAGAAAAACGCTGAGCAAAAAGGATTAAACACAGAAAAATTAAGAATTAAACACATATC
ATCAAACAGAGGCTTCACAATTAA**AAGAT**ATA**TGCCAAGA**GCATTTGGTAGAGCAAGCCCTAAAAAC**CAA**
GAAACAATACATATCCAAGTAATCCTTGAAGAATTCTACTAA

7 1.5% 458 **3.6 (1.9) 1.8**

Methanococcus maripaludis strain OS7mut1 chromosome, complete genome NCBI Reference Sequence: NZ_CP020120.1: 1575149-1576153 L3

ATGGGT**ATGAA**AAAAAATAGACCTCGTAGAGGTTCCCTAGCATTTAGCC**CAAGA**AAAAGAGCTAAGAAAT
TAGTTCCTAAAATCAGATCATGGCCTGCAGATAAAAAAGTAGGGCTCCAAG**CATTC**CCTGTATACAAAGC
AGGAACAACACATGCTTTATTGATCGAAAACAACCCTAAAAGTCCAAACAGCAGTCAGGAAGT**ATTCA**CA
CCCGTAACTGTATTAGAAACACCGGACGTTACCGTAGCAGGAATCAGACTCTACGAAAAAACAACCAAAG
GGTTAAAAGCATTAACTGAAGTATGGGCTGAACAGTTAGATAGCGACTTAGGAAGAAAATTAACTTTAGT
TAAGAAAGAAGAGAAAAAAACTGTTGACGCTTTAGACGCAGTTTTAGAAAAAGCTACAGAAGTAAGAGCT
ATTGTTCACACAAACCCAAAAACTACAGGAATTCCAAAGAAAAAACCTGAAGTTGTTGAAATTAGAATCG
GTGGATCATCAGTTGCTGA**AAGAT**TAGCTTATGCTAAAGAAATCTTAGGCAAAACACTCGCAATTAGCGA
TGTTTTCGAAGCTGGCGAAATTATCGATACCTTAGCAATTACAAAAGGTAAAGGATTCCAAGGATCAGTT
AA**AAGATG**GGGAATCAAAG**TTCAA**TTTGGAAAACACCAAAGAAAAGGTGT**TGGTA**GACACACAGG**TTCAA**
TCGGCCCATGGAGACCTAGAAGAGTTATGTGGACTGTA**CCATT**ACCTGGTCA**AATGG**GTTTCCACCAAAG
AAC**TGAAT**ACAACAAAAGAATCTTAAAATTAGGCAGCGAAGGCGCTGAAATTACACCTAAAGGCGGATTC

TTAAACTACGGTGCTGTTAAAAACGGCTACGTTGTAGTTAAGGGAACTG**TTCAAG**GTCCTGCAAA**AAGAT**
TAGTAGTTTTAAGAGGATCTGTAAGAGCTGCTGAAGACAAATTCGGCTTACCTGAAATTGCTTACATCAG
TACAGAATCCAAACAAGGAAACTAA

9 1001 **7.8 (2.8) 0.4**

Methanococcus maripaludis L4

ATGAATGTTAAAGTTTACAATTT**AGATGGT**TCTGAAAAGGGAGATAT**TGAAT**TACCCGCTGTATTTGAAGCAGAA
TACAGGCCTGATTTAATTAAAAGGGCAGTAATCTCATCATTAACCGCAAAATTACAACCAAAAGGTTGCGATGCA
TTTGCAGGATACAGAACTTCAGCAAAATCAATCGGAAAAGGACACGGTAAAGCTAGAGTTAGAAGAACCGCACAA
GGTGCTGGTG**CATTC**GTACCCCAAGCAGTTGGTGGAAGAAGAGCTCACCCTCCTAAAGTTGAAAAAATCTTATTC
GAAAGAATAAACAGAAAAGAAAAATTAAAAGCATTAGCAAGTGCTATTGCAGCTTCAGCAAACCCTGAAATCGTT
TCAGCAAGAGGCCACAAAATTGAAGGTGTTCCTTCATTACCTTTGGTTGTTAACGCAGAATTTGAGAGCCTTGTT
AAAACAAAAGAAGTTTTAGAAGTT**TTCAA**AACTTTAAAATT**AGATG**CTGACTTAGAAAGAGCTAAAGACGGCGTT
AAAATTAGAGCAGGAAGGGGAAAATTAAGAGGAAGAAAATACATAAAACCAAAAAGTGTTTTGGTTGTTGTAGGC
GATGTATGTGAAGCAAT**TACTGC**A**TCAAGA**AACCTCGCAGGTGT**AGATG**TAATTACAGCTAACGATTTAAGCGCA
ATACACATTGCACCAGGAAC**AATGG**CTGG**AAGAT**TAACACTCTGGACTGAAAA**TGCCATT**GAAAAAATAAACGG**A**
AGATTTTAA

9 755 **5.9 (2.4) 1.3**

Methanococcus maripaludis L1

ATGGACAGTGAAAAAA**TACTG**AACGCAGTGAAGGAGGCTCGAACTCTTGCTAAGCCGCGAAACTTCACGCAATCC
GTTGATCTCATCGTCAACTTAAAAGAGTTGGATCT**TTCAAGA**CCTGAAAACAGGTTGAAAGAGCAAATCGTTTTA
CCAAGTGGAAGAGGTAAAGACGTAGCGATTGCGGTTATTGCGAAAGGTGACTTGGCTGCTCAAGCAG**AAGAT**ATG
GGCCTCACTGTAATAAGACAGGAAGAATTAGAAGAATTAGGTAAAAACAAAAAAACAGCTAAAAAAATTGCTAAC
GCACACGGCTTCTTTATCGC**TCAAG**CTGATATGA**TGCCATT**GGTCGGTAAATCATTAGGTCCGGTTCTAGGTCCT
AGAGGTAAAA**TGCCA**CAACCAGTGCCTGCAAATGCAAACTTAGCTCCATTAGTTGCTAGATTCCAAAAAACCGTT
GCAATAAACACCAGGGATAAAGCATTATTCCAGGTATACATCGGAACAGAATCAATGAGCGAC**GATGAA**CTTGCT
GCAAATGCCGAAGCAATTTTAAACGTAGTGTCTAAAAAATACGAAAAAGGACTCTACCACGTTAAAAATGCATTT
ACAAAACTTAC**AATGG**GCGCAGCTGCTCCTATTGAGAAATAG

6 638 **5 (2.23) 0.45**

Methanococcus maripaludis strain OS7mut1 chromosome, complete genome NCBI Reference Sequence: NZ_CP020120.1: 1464911-1465375 L30

ATGGCTTACGCAGTTGTAAGAGTCAGAGGAAGCGTAGGTGTTAGAGGAGACATCGCAGATACC**ATGAAGA**
TGTTAAGACTACACCGTGTAAACCACTGTGTTATAATTCCAGATACAGAACACTACACTG**GAATG**ATAAA
AAAAGTTAAAGACTATGTAACCTACGGTGAAATCGATA**AAGAT**ACACTTGTAGCTTTAATTTTAAAAAGA
GGAAGACTTCCTGGAAACAA**AAGAT**TATCTGAAGAACTCGTAAAAGAGTTAACAGAACTTCCAGTTGAAG
AGTTAGCTGAAAAAGTAATTGCTGGCGAAATTAAAATAAAAGACACACCAATAAAACCTGT**ATTCA**GATT
ACATCCTC**CAAGA**AAAGGATATGATAGAGCAGGAGTTAAAAAAGGATTCTCAATTGGTGGTGCCTTAGGT
TACAGATC**TGGTA**AAATCAACGATTTATTGAACAAAATGATGTAA

4 461 **3.6 (1.9) 0.2**

Homo sapiens ribosomal protein S12 (RPS12), mRNA NCBI Reference Sequence: NM_001016.4

CTCTTTCC**CTGCC**GCCGCCGAGTCGCGCGGAGGCGGAGGCTTGGGTGCG**TTCAAGATTCAA**CTTCACCCG
TAACCCACC**GCCAT**GGCCGAGGAAGGCATTGCTGCTGGAGGTGT**AATGG**ACGTTAA**TACTGC**TTTA**CAAG**
AGGTTCTGAAG**ACTGCC**CTCATCCACGATGGCCTAGCACGTGGAATTCGCGAAG**CTGCCA**AAGCCTTAGA
CAAGCGCCAAGCCCATCTTTGTGTGCTTGCATCCAACTGT**GATGA**GCCTATGTATG**TCAAG**TTGGTGGAG
GCCCTTTGTGCTGAACACCAAATCAACCTAATTAAGGTT**GATGA**CAA**CAAGA**AACTAGGA**GAATGG**GTAG
GCCTTTGTAAAATTGACAGAGAGGGGAAACCCCGTAAAGTGGTTGGTTGCAGTTGTGTAGTAGTTAAGGA
CTATGGCAAGGAGTCTCAGGCCAAGGATGTCATTGAAGAGTAT**TTCAA**ATG**CAAGA**A**ATGAA**GAAATAAA
TCTTTGGCTCACA

13 499 **3.9 (2) 4.6**

Homo sapiens ribosomal protein S17 (RPS17), transcript variant 1, mRNA NCBI Reference Sequence: NM_001021.6

GTTTCCTCTTTTACCAAGGACCCGCCAACATGGGCCGCGTTCGCACCAAAACCGTGAAGAAGGCGGCCCG
GGTCATCATAGAAAA**GTACT**ACACGCGCCTGGGCAACGACTTCCACACGAACAAGCGCGTGTGCGAGGAG
ATC**GCCATT**ATCCCCAGCAAAAAGCTCCGCAA**CAAGAT**AGCAGGTTATGTCACGCATCT**GATGAA**GCGA**A**
TTCAGAGAGGCCCAGTAAGAGGTATCTCCA**TCAAG**CTGCAGGAGGAGGAGAGAGAAAGGAGAGACAATTA
TGTTCCTGAGGTCTCAGCCTTGGATCAGGAGATTATTGAAGTAGATCCTGACACTAAGGAAATGCTGAAG
CTTTTGGACTTCGGCAGTCTGTCCAACCTTCAGGTCACTCAGCCTACAGTTGG**GATGAAT****TTCAA**AACGC
CTCGGGGACCTGTT**TGAAT**TTTTTCTGTAGTGCTGTATTATT**TTCAA**TAAATCTGGGACAACAGCCTT

8 1.7% 484 **3.8 (1.9) 2.2**

Homo sapiens ribosomal protein S9 (RPS9), transcript variant 1, mRNA NCBI Reference Sequence: NM_001013.4

CTCTTTCTCAGTGACCGGGTGGTTTGCTTAGGCGCAGACGGGGAAGCGGAGCCAACA**TGCCA**GTGGCCCG
GAGCTGGGTTTGTCGCAAAACTTATGTGACCCCGCGGAGACCCTTCGAGAAATCTCGTCTCGAC**CAAGA**G
CTGAAGCTGATCGGCGAGTATGGGCTCCGGAACAAACGTGAGGTCTGGAGGGTCAAATTTACCCTGGCC**A**
AGATCCGCAAGGCCGCCCGGGA**ACTGC**TGACGCTT**GATGA**GAAGGACCCACGGCGTCTGTTCGAAGGCAA
CGCCCTGCTGCGGCGGCTGGTCCGCATTGGGGTGCTG**GATGA**GGG**CAAGATGAA**GCTGGATTACATCCTG
GGCCTG**AAGAT**AGAGGATTTCTTAGAGAGACGCCTGCAGACCCAGGTC**TTCAAG**CTGGGCTTGGCCAAGT
CCATCCACCACGCTCGCGTGCTGATCCGCCAGC**GCCAT**ATCAGGGTCCGCAAGCAGGTGGTGAACATCCC
GTCCTTCATTGTCCGCCTGGATTCCCAGAAGCACATCGACTTCTCTCTGCGCTCTCCCTACGGGGGTGGC
CGCCCGGGCCGCGTGAAGAGGAA**GAATG**CCAAGAAGGGCCAGGGTGGGGCTGGGGCTGGAGACGACGAGG
AGGAGGATTAAGTCCACCTGTCCCTCCTGGGCTGCTGGATTGTCTCGTTTTC**CTGCCA**AATAAACAGGAT
CAGCGCTTTACAA

9 709 **5.5 (2.35) 1.49**

Homo sapiens L3 mRNA, complete cds GenBank: AY598322.1

ATGATGGCCACTCCGAACCAGACCGCCTGTAATGCAGAGTCACCAGTGGCCCTGGAGGAGGCCAAGACCT
CTGGTGCCCCGGGGAGCCCCCAAACACCCCCTGAGCGTCATGACTCTGGTGGTTCC**CTGCC**CCTGACACC
GCGGATGGAGAGCCACTCAGAG**GATGAA****GAT**CTTGCTGGGGCTGTCGGTGGCCTGGGCTGGAACAGTAGG
AGTCCCCGGACCCAGAGCCCAGGGGGCTGCTCAGCGGAGGCTGTGCTGGCCCGGAAGAAACACCGTCGGC
G**GCCAT**CGAAGCGCAAAAGGCACTGGCGACCCTACCTGGAGCTGAGCTGGGCTGAGAAACAACAGCGG**GA**
TGAGAGGCAGAGCCAGAGGGCCTCCCGGGTCCGCGAAG**AGATG**TTCGCCAAAGGCCAGCCCGTGGCCCCC
TACAACACCACCCAGTTCCT**GATGAATG**ACAGGGACCCGGAGGAGCCCAACTTGGATGTGCCCCATGGGA
TCTCCCACCCAGGTTCCAGTGGGGAGAGTGAGGCCGGGGACAGTGATGGGCGGGGCCGAGCGCACGGTGA
GTTCCAGCGGAAGGACTTCTCTGAGACTTACGAACGCTTCCACACCGAGAGCCTGCAGGGCCGCAGCAAG
CAGGAGCTGGTGCGAGACTACCTGGAGCTGGAGAAGCGGCTGTCGCAGGCGGAGGAGGAGACTAGGAGGC
TGCAGCAGCTGCAGGCGTGCACCGGCCAGCAGTCCTGCCGCCAGGTGGAGGAGCTGG**CTGCC**GAGGTCCA
GAGGCTCCGGACCGAAAACCAGCGGCTTCGTCAGGAGAACC**AGATG**TGGAACCGAGAGGG**CTGCC**GCTGT
GATGAGGAGCCGGGTACCTAG

9 1.3% 657 **5.1 (2.3) 1.7**

Homo sapiens ribosomal protein L22 (RPL22), mRNA NCBI Reference Sequence: NM_000983.4

CCTTTCTAACTCCGCTGCC**GCCAT**GGCTCCTGTGAAAAAGCTTGTGGTGAAGGGGGGCAAAAAAAAGAAG
CAAGTTCTGAAGTTCACTCTTGATTGCACCCACCCTGTAG**AAGATG**GAATCATGGATG**CTGCCA**ATTTTG
AGCAGTTTTTG**CAAGA**AAGGATCAAAGTGAACGGAAAAGCTGGGAACCTTGGTGGAGGGGTGGTGACCAT
CGAAAGGAG**CAAGA**G**CAAGAT**CACCGTGACATCCGAGGTGCCTTTCTCCAAAAGGTATTTGAAATATCTC
ACCAAAAAATATTTGAAGAAGAATAATCTACGTGACTGGTTGCGCGTAGTTGCTAACAGCAAAGAGAGTT
ACGAATTACGTTACTTCCAGATTAACCAGGACGAAGAAGAGGAGGAAGACGAGGATTAAATTTCATTTAT
CTGGAAAATTTTGTATGAGTTCTTGAATAAAACTTGGGAACCAA**AATGGT**GGTTTATCCTTGTATCTCTG
CAGTGTGGATTGAACAGAAAATTGGAAATCATAGTCAAAGGGCTTCCCTTGGTTCGCCACTCATTTATTT
GTAACTTGACTTCTTTTTTTTTCTGCTTAAAAAT**TTCAA**TTCTCG**TGGTA**ATACCAGAGTAGAAGGAGAG
GGTGACTTTACCGAACTGACA**GCCATT**GGGGAGGC**AGATG**CGGGTGTGGAGGTGTGGGCTGAAGGTAGTG
ACTGTTTGATTTTAAAAAGTGTGACTGTCAGTTGTATCTGTTGCTTTTCTCAATGATTCAGGGATACAAA
TGGGCTTCTCT**CATTCA**TTAAAAGAAAACGCGACATCTTTCT**AAGAT**TCTCTGTGGGAAAATGACTGTCA
ATAAAATGCGGGTTTCTGG**GCCATTC**GTCTTACTTTCATTTTTTGATTACAAATTTCTCTTGACGCACAC
AATTATGTCTGCTAATCCTCTTCTTCCTAGAGAGAGAAACTGTGCTCCTTCAGTGTTG**CTGCCAT**AAAGG

GGTTTGGGGAATCGATTGTAAAAGTCCCAGGTTCTAAATTAACTAAATGTGTACAGAA**ATGAA**CGTGTAA
GTAATGTTTCTACAGGTCTTTGCAACAAACTGTCACTTTCGTCTCCAGCAGAGGGAGCTGTAGGAATAGT
GCTTCC**AGATG**TGGTCTCCCGTGTGGGGCCCAGC**AATGG**GGGCCCCTGATGC**CAAGA**GCTCTGGAGGTTC
TTGAAAGAGGGGACACGAAGGAGGAGTGACTGGGAAGCCTCCCA**TGCCA**AGGAGGTGGGAGGTGCCCTGG
AAATAGCTGCCTCA**TGCCA**CTTAG**GCCAT**GACTGGATTTAATGTCAGTGGTG**TGCCA**CAGTGCAGAGGCT
AGACAACTGAAAGGGGCTACCAAGGCTGGGAAAAAAATGCAATTGTTGCTGTGAGTGACTTTGAAAGACT
CTGGTGCCTTGTGGTGCCCTTCTGAA**ATTCAA**ACAGTAATGCAAAAGTGTCTGCATTAGAATTTACGGTG
TCTAAA**ATTCA**TGTTTTTAAAAGAGCTTGCCTAC**AGATG**GTTTCCACACTTGAAATTGTGCCCTGCGAGT
TGCATAGCTGGAAG**TTCAA**TGCTCAGTCCTACCTTGGCTCCCATTAAACATTTGGTGCTCTGTGGATTGA
GTTGAACGTGTTGAGGCTTTGCAATTTCACTTGTGTTAAAGGCTCTGGCATTTTT**CCATT**TCTATGCAAA
TTTCTTTGAAGCAGAATTGCTTGCATATTTCTTCT**CTGCC**GTCACAGAAAGCAGAGTTTCT**TTCAA**ACTT
CACTGAGGCATCAGTTGCTCTTTGGCAATGTCCCTTAACCATGATTATTAACTAAGTTTGTGGCTTGAGT
TTACAAATTCTACTTGTTGCATTGATGTTCCCATGTAGTAAGTCATTTTTAGTTTGGTTGTGAAAAAACC
CTGGGCTGAAGTTGGCATTTCAGTTAAAAGAAAAAAAGAAACTAGTCCCAGATTTGAAAACTTGTAATAA
AATTGAAACTCACTGGTTTTCTATGTCTTT**TTGAA**CTCTTGTAATCGAGTTTTGATCATATTTTCTATTA
AAGTGGCTAACACCTGGCTACTCT**TACTG**TA

16 0.7% 2057 **16 (4) 0**

Homo sapiens L4 mRNA, complete cds GenBank: AY598323.1

ATGTTTGGTTTTCACAAGCCA**AAGATG**TACCGAAGTATAGAGGGCTGCTGTATTTGCAGAGCTAAGTCCT
CCAGTTCTCG**ATTCA**CTGACAGTAAACGCT**ATGAA**AAGGACTTCCAGAGCTGTTTTGGATTGCATGAGAC
TCGTTCAGGAGACATCTGCAATGCCTGTGTCCTGCTTGTGAA**AAGATG**GAAGAAGTTGCCAGCAGGATCA
AAAAAAAACTGGAATCATG**TGGTAGATG**CAAGGGCTGGACCCAGTCTAAAGACTACATTGAAACCAAAGA
AAGTGAAAACTCTATCTGGGAACAGGATAAAAAGCAACCAGATCAGTAA**ACTGC**AGAAGGAATTTAAACG
TCATAATTCTGATGCTCACAGTACCACC**TCAAG**TGCCTCCCCAGCTCAATCTCCTTGTTACAGTAACCAG
TC**AGATGA**CGGCTCAGATACAGAGATGGCTTCTGGTTCTAACAGAACACCAGTTTTTTCCTTTTTAGATC
TCACTTACTGGAAAAGACAG**AAGAT**ATGTTGTGGGATCATCTATAAAGGCCGTTTTGGGGAAGTCCTCAT
TGACACACATCTC**TTCAAG**CCTTGCTGCAGCAATAAGAAAGCAGCTGCTGAGAAGCCAGAGGAGCAGGGG
CCAGAGCCT**CTGCC**CATCTCCACTCAGGAGTGGTGA

10 662 **5.17 (2.3**) **2.1**

PREDICTED: Homo sapiens mitochondrial ribosomal protein L1 (MRPL1), transcript variant X2, mRNA NCBI Reference Sequence: XM_047416090.1

GCAATCCGGAGTGCCCAACATGGCGGCGGCCGTAAGGTGCATGGGTAGAGCCTTGATACATCATCAAAGG
CATAGCCTTTC**CAAGATGGT**TTATCAGACATCACTTTGTTCTTGTTCTGTAAACATCCGAGTGCCCAACA
GACATTTTGCTGCTGCTACAAAGTCTGCAAAGAAAACAAAAAAAGGTGCTAAAGAAAAAACACC**AGATGA**
GAAAA**AAGATGAA**ATAGAAAAAATAAAAGCATATCCCTATATGGAAGGCGAACCTGAG**GATGA**TGTCTAT
TTAAAACGCTTATACCCGAGACAGATATATGAGGTGGAGAAAGCTGTTCACTTACTTAAGAAAT**TTCAA**A
TTCTTGACTTTACTAGTCCAAAGCAAAGTGTTTATCTTGATTTGACACTGGATATGGCACTGGGAAAGAA
GAAAAACGTGGA**GCCATT**TACCAGTGTTCTTAGTT**TGCCAT**AC**CCATT**TGCTTCCGAAATCAATAAAGTT
GCTGTATTTACAGA**GAATG**CATCAGAGGTCAAAATAGCGGAAGAA**AATGG**AGCTGCATTTGCAGGAGGCA
CTAGTCTGATACAG**AAGAT**TTGGGAT**GATGAA**ATTGTTGCAGACTTTTACGTAGCTGTTCCAGAAATAAT
GCCTGAACTTAATCGATTAAGGAAGAAAC**TGAAT**AAAAAATATCCAAAGCTTTCTCGAAATTTTAA**ACTG**
CCTATGGGTAGTTGCTTTCACTTATAGCTGTAGTGCTCCTGAGCCAACTGAACAACTGCTGAGATAATCC
TAGGAATAATGTATGCTGAGGGTATGCCGTGCTCTCTATGTAATGAGTTCCTCACATTG**ATTCA**TCTATG
GAAGACTTGCTTGAGC**ATGGT**GGGAGTGATGTCTCTTCAGTGGCACAGTCTT**CATTC**TATTTCTTACCCT
TTAAATAATTCACATTTTTCAGTGCATGGAATTAAATTTT

14 946 **7.4 (2.7) 2.4**

Homo sapiens ribosomal protein L30 (RPL30), mRNA NCBI Reference Sequence: NM_000989.4

CCTTTCTCGTTCCCCG**GCCAT**CTTAGCGGCTGCTGTTGGTTGGGGGCCGTCCCGCTCCTAAGGCAGG**AAG**
ATGGTGGCCGCAAAGAAGACGAAAAAGTCGCTGGAGTCGATCAACTCTAGGCTCCAACTCGTT**ATGAA**AA
GTGGGAAGTACGTCCTGG**GGTAC**AAGCAGACTCTG**AAGATGA**TCAGACAAGGCAAAGCGAAATTGGTCAT
TCTCGCTAACA**ACTGCC**CAGCTTTGAGGAAATCTGAAATAGA**GTACT**ATGCTATGTTGGCTAAAACTGGT
GTCCATCACTACAGTGGCAATAATATTGAACTGGGCACAGCATGCGGAAAAATACTACAGAGTGTGCACAC

TGGCTATCATTGATCCAGGTGACTCTGACATCATTAGAAGCATGCCAGAACAGACTGGTGAAAAGTAAAC
CTTTTCACCTACAAAATTTCACCTGCAAACCTTAAACCTGCAAAATTTTCCTTTAATAAAATTTGCTTGT
TTTAAAAA

2 494 **1.9 (1.4) 0.7**

Table S5. Glycyl-tRNA ligase sequences from different species with after the sequence: Nb of observed pentamers among the pentamer set {ATTCA TTCAA TCAAG CAAGA AGATG GATGA ATGAA TGAAT} with percentage among the set of pentamers of the sequence (in red), Nb of nucleotides in the sequence (in black), Nb of expected pentamers with its standard deviation (in red), Nb of standard deviations between observed (in blue) and probability of this event (in green).

Acinetobacter baumannii strain ATCC BAA-1790 chromosome, complete genome GenBank: CP042841.1: c395610-393541 Glycyl-tRNA synthetase subunit beta

ATGAGCAAACATACTGTTTTATTCGAACTTGGCTGTGAAGAGCTTCCACCAAAAAGCTTAAAAACATTAC
GTGATGCACTACAAGCAGAAACTGTAAAAGGCTTAAATGAAGCAGGTTTAAACTTCGCATCTGTTGAAGC
TTATGCCGCTCCACGTCGTTTAGCGCTTAAAATTGTAGATGTAGATGCTGCTCAAGCGGATACACAAAAA
CGTTTTGACGGCCCTGCTGTTCAAGCAGCTTACGATGCAGAAGGCAAACCGACTAAAGCACTTGAAGGTT
TTATGCGCGGTCAAGGTATTACTGTTGATCAGCTTTCTACATTCCAAGCAGGTAAAGTTGAAAAAGTTTG
CTATTTAAAAGATGTGAAAGGCCAAAGCCTTGACACTTTGCTTCCACAGATTTTACAAACTGCGTTAGAC
AATTTACCAATTGCTAAGCGCATGCGTTCTGCTGCAAGCCGTACCGAATTTGTACGTCCAGTGAAATGGG
TTGTCTTACTCAAAGATGACCAAGTTATTGAGGCGACTATTCAAGATCACAAAGCTGGTAATGTGACTTA
TGGTCACCGTTTCCATGCACCTGAAGCTGTGACCTTAGCGCATGCAAATGACTATTTAGCTGCGTTAGAA
AAAGCATATGTTGTAGTGAACTTTGAAAAGCGTCAAACAACTATTCAAGAACAAGTGAAAAAGTTAGCTG
ATGAAGTGAACGCAACTGCGATTGTTCCAGCTGACTTACTTGATGAAGTAACAAGTCTGGTTGAATGGCC
TGTAGCTTTACGTGCAACTTTTGAAGAACGCTACTTAGCTGTTCCTCAAGAAGCGCTCATTACGACAATG
CAAGACAACCAGAAGTATTTCTGTTTGATCAATGCGGAAGGTAAATTACAGCCTTACTTCATTACTGTTT
CAAATATTGAGTCTAAAGACCCGACTCAAATTATTGAAGGTAACGAAAAAGTTGTTCGCCCTCGTTTGTC
TGATGCTGAGTTCTTCTTCTTACAAGATCAAAAGCAACCGCTTGCATCTCGTAAAGAAAAATTAGCAAAC
ATGGTATTCCAAGCACAGCTTGGTACGCTTTGGGACAAATCAACACGTATTGCAAAACTTGCAGTTGCAT
TATCTTCAATCACTGGTGCAAACCCAGCGGATGCTGAAAAAGCTGCATTACTTGCTAAATGTGACTTAAC
TTCTGAACTTGTAGGTGAGTTCCCCGAACTTCAAGGTATTGCGGGTACTTACTACGCGCGTATTGAAGGC
GAAAACACTGAAGTTTCAGAAGCTTTAGGTGAACAATACTTACCAAAATTTGCGGGTGATGTTTTACCTA
AAACCAAAACAGGTACAACCATTGCCCTTGCTGACCGTTTAGATACGCTTGTTGGTATTTTCGGTATTGG
ACAAGCGCCTACAGGTTCTAAAGACCCATTTGCTCTTCGCCGTTCAGCAATTGGTATCTTACGCTTAATC
ATTGAAAATGAATTAGATGTAACAATTGAAGAGCTAGTTAATCTTGCACTTCAAGGTTATGGCGATATCG
TTAAAGACCACGACAAAACTCGTGCTGATGCGGTTGCATTCTTGGAAGGTCGTTACCGTGCTAAGTATGA
AGACCAAGGCGTTGCTGTTGACGTCCTTCAAGCAGTTCAAGCTCTTGCACCAAAATCTCCACTTGATTTT
GACAAGCGCGTAAATGCTGTTAACCACTTCCGTACATTACCTGAAGCTGCTGCACTTGCTGCTGCAAACA
AACGTGTTGCTAACATTCTTGCAAAAGAAACAGCACCAGAAGGTTCTGTCGTTGAAGCGAACTTGGTTGA
AGATGCTGAAAAAGCATTATTTGCTGAGCTTCAAGCAGTGACACCTGTGGTTGAACCATTACTTGCAGCA
AAAGACTACACAGCTGCCCTTTCTAAGCTTGCAGCACTTCGCGCGCCGATTGATGCATTCTTTGATGGTG
TAATGGTAATGGCAGATGATGCTGACCTAAAAGCAAACCGTTTACGTTTATTAGCTCAGTTACGTAACTT
GTTTACTGCTGTGGCTGATGTGAGTGTGTTGCAGGGTTAA

44 2.1% 2066 **16.1 (4) 7 3** **10^{-13}**

Striga asiatica UVA1 DNA, scaffold5, whole genome shotgun sequence GenBank: BKCP01008292.1 glycyl-tRNA synthetase

ATGAATGTCGCTCAAGAAGCCCAGAACCGGGAGGCATTCCGGCACGCCGTCGTCAACACCCTCGAACGCC
GTTTGTTTTACATACCTTCCTTCAAGATCTACCGCGGTGTCGCCGGCCTTTATGATTATGGGCCTCCGGG
CTGCGCCGTTAAGTCTAACGTCCTCGCCTTCTGGCGTCAGCATTTTGTCCTGGAGGAGAACATGCTAGAA
GTTGATTGCCCATGTGTCACTCCAGAGGTTGTGCTCAAGGCATCAGGCCACGTGGACAAGTTCACCGACC
TTATGGTCAAAGACGAAAAGACTGGAACATGTTACCGAGCAGATCATCTGCTCAAGGATCATTGCAAAGA
AAAGCTCGAGAAGGACCCTAATCTCAGTGCTGATAAGGCAACTGAACTTAGGCATGTGCTTGCTGTGCTG
GATGATCTTTCTTCAGAGGAGCTTGGTGCTAAAATAAAGAGTTATGGAATTACTGCTCCGGACACAAAGA
ACAGTCTGTCAGACCCTTACCCTTTTAACCTAATGTTTCAGACATCAATTGGGCCATCTGGCTTGAGCAG
TGGGTATATGCGTCCTGAGACGGCTCAGGGTATTTTTGTGAATTTCAAGGACTTGTACTACTATAATGGC
AACAAGCTTCCTTTTGCAGCTGCACAGATTGGTCAAGCTTTCCGAAATGAGGTCTCGTATTCTACCATGA
TGCTTGCATTGCTACTTGCTTTCTTTGAGTATCTTCCTAGTGTAGCCTGGGACCTGGAGAAGATCTCTCC
TAGGCAAGGTCTACTTAGGGTCCGTGAATTCACACTGGCAGAGATTGAGCACTTTGTGGATCCTGAGGAT

AAGTCTCACCCTAAATTCGCTGAGGTGGCACAATTGGAATTCTTAATGTTCCCAAGAGAAGATCAGGTGT
CTGGACGAGCGGCTAGGAGAATGGCTATTGGGGAAGCAGTTGAACAGGGGATTGTGAACAATGAGACACT
TGGTTATTTCATCGGGCGTGCGTATTTATTCTTGACCCAACTTGGGATTGACAAAGACCGATTAAGGTTT
CGCCAGCATCTGGCAAATGAGATGGCACACTATGCTGCGGATTGTTGGGATGCTGAAATCGAATGCTCTT
ATGGTTGGATCGAGTGTGTTGGTATTGCTGATAGATCAGCATATGATCTGCGTGCCCACACTGATAAAAG
TGGAGTTCCCCTTGTGGCACATGAGAAATTCACAGAACCTAGAGAAGTCGAGAAGCTTGTTATAGCTCCA
GTTAAGAAAGAGCTGGGCCTTGCCTTTAAGGGTAGCCAAAAGATGGTGGTTGAAGCTTTGGAGGCCATGG
ATGAAAAGGAAGCCATGGAGTTGAAAGCAACTCTGGAAGCCAAAGGAGAGGCCTCGTTTCGTGTTTGCAC
TCTTGAAAAGACCGTGAACATAAAGAGTAATATGGTCTCCATCTCCAAGAAGAAGAAAAAGGAGCACCAG
AGGGTTTTCACTCCATCGGTTATCGAGCCATCGTTCGGTATTGGGAGGATCATATATTGCCTCTATGAGC
ACTCGTTTTACACGAGGCCTAGCAGAGATGGAGATGAACAGCTCAATGTCTTTGGCTTCCCGCCTCTTGT
AGCCCCCATCAAATGCACTGTTTTTCCTCTGGTTCAGAATGAGCAATTTGAGGAAGTTGCTAGAAGGATT
GCCAGGTCACTAACTGCCGTTGGGATCACGTACAAAATCGACATTACTGGAACCTCGATTGGGAAGAGAT
ATGCAAGAACGGATGAACTGGGAGTCCCGTTAGCAATCACTGTTGACTCAGAAAGCTCGGTGACTGTGAG
AGAGAGGGACAGCAAGCAGCAGATTCGTGTTAGTGTTGATGAGGTGGCATCTGTGGTGAAGGAGTTTACA
GATGGAGTGAGCACCTGGGGCGACGTGCTATGGAGATACCCCACTCATTCAAATTGTGATGACGTGTCGT
GGATTGTCCAAAAACTTTGCTATGAATCAGATCGCAGATATGAGGCCCAATATCGTATTCCTCAAGGCCT
TCTCTCGCTACTGAAATTTCTTTCCGATAAGGTCATCCCGGACATCAACTCACCGGCCAGACATCTGCCG
CCGGCACTCGGGCCGCCAACGGTTGTGAAAAGCATGGTAGTTTTGAAAAGAGAACTATTTTATCTGAGAT
CATCATTTCGTCGGAGCCAAACTGCAGTTTTCTGCACAAAACCTGAGCGCACTTTTGCTTCATCGCAAGC
TCTTTCCGTAACAGATGATCAAACACTTTTCAGCATTCTTGAATCTTGCAAGCTGTCCCACAATTTCAGA
ACTGCAAATACTGTGCACAGTAGAATCATTAAACATGGGTATGGAATGTACCCATTGCTATTATCCACGT
TACTGAAGGTGTACATGGCTTGCCAAAAACCCATTCTTGCTCAACAACTACTTGCCGAGGTATGTTGTCC
AAATTTTGGCATCTCTGCAAATTTGCTGATTGATAGTTGTTTGAAGACTGGCGAAATAGATATGGCAGAG
AAAGTATTTTCTGCTCTACCTATCCGAGATGTCGTCACATGGAACTTGATCATTGGGGGTAATGTCAAAA
GTGGATTCTTTGTGGAAGCAATTGAGTTGTTTAAACAAATGTTAGAAACAAATATTGAGCCAGATGGGTT
TACATTTGCTTCAGTCATAAACGCGTGCTCAAGGCTTGGAGCTCTTAGCCATGGAAAGTGGGTGCATAGT
TTGATGATCGAGAAAAAAATCGAGTTAAATTATATACTTTCTTCTGCACTTATAGACATGTACTCTAGAT
GTGGTAGAATTGAAACAGCAAAATCCATCTTTAGCAATGCTCAGAAAACGGATGTATCCATATGGAATGC
AATTATAAATGGACTAGCAATTCATGGGCTTGCTGTGGATGCAATTTCTGTTTTCTCAGCCATGGAAAGG
GAAAAAGATATTTCACCTGATTCTGTAACCTTTATGGGAATCATAAAGGCTTGTAGCCACTGCGGCTTAC
TAGAACAGGGTCAATGGTATTTTAATCTAATGCAAAAAAGTTACTTGATTCGGCCGGAAATCGAGCACTA
TGGGGCAATGGTCGATCTTTTCGCACGAGCCGGACTTCTGGAAGAAGCTTATGCAATGATTAATGAGATG
CCAATGGAGCCTGATGTTTTTGTATGGAGGACACTACTTAGTGCATGTAGAACTCACAAAAAATCCAAGT
TGGGTGAAGAAGTTGTCGGGAAAATAAAGCATTTAGGCAGTGGGGATTATGTTTTGATGTCAAATATGTA
CTGCTCAACAAAGAAATGGGATTATGCAGAAAATATGAGGCAAACTATGAATAAGAAGGGAGTTCATAAG
GCTAAGGGAAAGAGTTGGGTTGAACTGCATGGTATGATTCACCATTTTAGGGCAGGTGATAGGTCCCACC
CAGAAACCGAATCAATTTATACACTATTGGAAGCTTTGACTCGTCGATCTAGAATGGAAGGTTATGTTTC
TGTACAGGATTTGGTTTTGATGGATATATCAGAAGAAGAAAAGGAGGAGAACTTGAATTATCACAGTGAA
AAGTTGGCTGTGGCTTGCGGGATCCTGAAATCTAGTCCTGGAACTGAAATATTGATTTCCAAAAATTTGC
GAACTTGTCTTGATTGTCACAGTTGGATCAAGATTGTGTCGAAAGTTCTGCATAGGGTGATAAACATGAG
GGACAGGATTCGCTTTCATAGGTTTGAGAAGGGCTCTTGTTCTTGTGGTGATTATTGGTAA

62 1.6% 3768 29 (5.3) 6.1 10^{-9}

Sugiyamaella lignohabitans glycine--tRNA ligase (GRS1), partial mRNA NCBI Reference Sequence: XM_018879933.1

ATGTCCGAATCAACTATTACTTCCAGACCCCCAGTTCAGGATCGAGAAGTCCTTGAATCTCTGCTGAAAC
ACAGATTTTTCTACGGTCCTTCTTTCGAAATATACGGCGGTGTTTCTGGATTGTACGATTACGGTCCAGC
TGGTTGCGCCTTACAAGCCAACATTATCGACACTTGGCGTAAACACTTCGTGCTTGAAGAAGATATGCTT
GAGGTCGATTGTCCTGCTTTGACTCCCTACGATGTTCTTAAGACTTCTGGTCACGTTGATAAATTTGCCG
ATTGGGTTTCTCGTGATTTGGCTACTGGTGAAATCTTCCGTGCGGATCACTTGGTCGAGGAAGTGCTTGA
AAGCCGCCTCAAGGGTGATAAGGAAGCTCGTGGTGTCGTAGAATCAACCGACCCTGCCAAGGAAGAAAAA
GACAAAAAGCGCAAGAAGAAGGTTAAGGAGACCAAGGCTATCAAACTTGATGATGAAACTGTTGCTGAGT
ATGAGTCAATCTTGGCCCAAATTGATGGCTACTCTGGCCCTCAATTGGGTGAGTTGATGGTAAAGCACAA
GATTGTTAATCCTACCACTGGTGGAGAATTGGAAGCTCCAAGGGAATTCAATCTTATGTTTGAGACAGCC
ATTGGCCCCTCTGGTCAATTGAAAGGTTATCTTAGGCCTGAAACTGCCCAGGGTCAATTTTTGAACTTCT
CCAAACTACTTGAAAGTAACAACTCCAAGATGCCATTCGCATCAGCTTCTATTGGTAAATCATTTAGAAA
CGAGATTTCTCCTCGTGCTGGTTTACTTCGTGTTCGAGAATTTTTGATGGCCGAAGTCGAACATTTCGTC
GACCCTAACGATAAATCTCATGCTCGTTTTGATGAAATTAAGGATATTCGATTGAGATTATGGCCCATGG
ATGTTCAAGCTCAAGGTAAAACTGATATCAGCGAACTTTCTATCGGCGAAGCTGTTTCAACTGGTATGAT

TGACAACCAAACTCTCGGTTATTTCCTTGCTCGAATTTACCTCTTCATGATT**AAGAT**CGGTATCGATCC**C**
AAGAGAATGAGATTTAGACAACACATGGCCAATG**AGATG**GCACACTATGCTGCTGATTGTTGGGATTGTG
A**ACTGC**AATCTTCTT**ATGGT**TGGATTGAGTGTGTTGGATGTGCTGACAGATCTGCTTATGACTTGACTGT
GCACT**CTGCC**C**GTACT**AAAGAAAAGCTCGTTGTCCGTCAAACCCTCGACGAACCGGTTACCGT**TGAAT**CA
TTTGAGGTGGAGTTGCAAAAGAAGAAGTTTGGTCCTAAGTTCCGTAAGGATGCTCCTAAGGTGGAAGAGT
TCTTGCTTAGCCGTACA**GAATG**TGAACGTGAGAACCTCGCC**CAAGA**ACTCAAAGACACGGGTAAAATCGT
TTTTGAGGTTGCCGGAATTGATGG**CAAGAT**CGAACTTGACTCATCATTTATCACTATTGAAAAAC**GTACT**
AGAGTTGAACATATTAGAGAGTACACTCCTAATGTAATTGAACCCTCATT**TGGTA**TTGGACGTATTCTTT
ACTCACTAATTGAACACTCTTTCTGGACTCGTCCCG**AAGATG**CTCAACGTGGTGTTTTATCCTTCCCACC
AACTGTTTCTCCTACCAAGGTTCTTGTCGTCCCCCTTTCCAGCAATGCAGA**ATTCA**CTCCCATCGCTCGT
GAACTGGTTTCCAAGCTTAGACGTCTTGG**CATTCAA**TCCAAGGTCGACGACTCATCCGCCTCTAT**TGGTA**
AGCGTTACGCTCGTAATGACGAGCT**TGGTACT**CCTTTCGGTATTACTATTGATTTCCAAACCG**TCAAG**GA
TGGTTCTTTAACTATTCGTGAACGTG**ATTCAA**CCAAGCAAGTCCGTGGCAG**TCAAGATGAA**GTGCTTGAG
GCACTCCGCTCTTTAATTTTTG**ATGGT**GTTTCCTGGGAGTCGGGAACATCACACCTTGCTACATTTGTCT
C**TCAAGAT**ATTGAGTAG

38 1.9% 1973 **15.4 5.8σ 3 10^{-9}**

Saccharomyces cerevisiae S288C glycine--tRNA ligase (GRS1), partial mRNA NCBI Reference Sequence: NM_001178469.2

ATGAGTGTAG**AAGAT**A**TCAAGA**AGGCTAGAGCCGCTGTT**CCATT**TAACAGAGAACAGCTAGAAAGTGTTT
TGAGAGG**AAGAT**TTTTTTATGCCCCAGCTTTTGACTTGTACGGTGGGGTTTCTGGTTTATATGACT**ATGG**
TCCACCAGGTTGTG**CATTC**CAAAACAACATCATTGACGCCTGGAGAAAGCATTTTATTTTGGAAG**AAGAT**
ATGTTGGAAGTTGATT**GTACT**ATGTTAACTCCATATGAGGTTTTGAAGACCTCAGGTCATGTTGACAAAT
TTTCTGATTGGATGTGTAGAGATTTGAAGACTGGTGAGATTTTCAGAGCTGA**CCATT**TAGTCGAAGAAGT
TTTGGAAGCTCGCTTAAAGGGTGAC**CAAGA**AGCCAGAGGTTTAGTCGAGGATGCTAATGCCGCTGCTAAG
GACGATGCCGAAAAGAAGAAGAGAAAGAAAAAAGTTAAACAAATTAAAGCAGTCAAATTG**GATGA**CGACG
TTGTCAAAGAAT**ATGAA**GAAATTTTGGCTAAAAT**AGATGGT**TATTCCGGCCCAGAATTAGG**TGAAT**TGAT
GGAAAAATACGATATCGGTAACCCAGTCACCGGAGAAACTTTAGAATCTCCAAGGGCTTTTAACCTGATG
TTTGAAACGGCTATTGGCCCATCTGGTCAATTAAAGGGTTACTTGAGGCCAGAA**ACTGCC**CAAGGTCAAT
TCT**TGAAT**TTTAACAAGTTGTTAGAATTTAACAACAGTAAGACCCCTTTTGCCTCAGCCTCTATCGGTAA
ATCATTTAGAAATGAGATTTCCCCAAGGGCCGGTTTATTAAGAGTCCG**TGAAT**TTTTGATGGCAGAGATT
GAACATTTCGTTGAC**CCATT**GGACAAATCTCATCCAAAATTTA**ATGAA**ATCAAAGACA**TCAAG**CTTTCCT
TTTTGCCTCGTGATG**TTCAAGA**AGCTGGTTC**TACTG**AACCAATTG**TCAAGA**CAGTTGGTGAAGCAGTTGC
TTCAAGAATGGTCGATA**ATGAA**ACTTTGGGTTACTTTAT**TGCCA**GAATTTACCAATTTTT**GATGAA**AATT
GGTGTT**GATGA**GTCCAAATTGAGATTCCGTCAACATATGGCTA**ATGAAATGG**CACATTATGCTGCTGACT
GTTGGG**ATGGTGAAT**TGAAGACATCTTACGGGTGGAT**TGAATG**TGTCGGATGTGCTGATAGAT**CTGCC**TA
TGATTTGACCGTT**CATTC**CAAAAAGACCAAAGAAAAATTGGTTGTTAGACAAAAATTAGATAATCCAATT
GAAGTCACCAAGTGGGAAATTGATTTGACGAAAAAATTGTTCGGTCCAAA**ATTCA**GAA**AAGATG**CACCAA
AAGT**TGAAT**CTCATCTAT**TGAAT**ATGTC**TCAAGATGA**CTTGGCTTCCAAGGCTGAGTTATTGAAGGCA**AA**
TGGCAAGTTTACTATAAAGGTTG**ATGGT**GTTGATGGCGAAGTCGAGTTGGACGATAAATTGGTTAAAATC
GAACAAAGAACAAAGGTTGAACACGTTAGAGAATATGTTCCTAGTGTCATTGAACCATCTTTCGGTATTG
GCCGTATCATCTACTCCGTTTTTGAGCACTCTTTCTGGAACAGACCAGAAGACAA**TGCCA**GAAGTGTTCT
TTCCTTCCCACCTTTGGTTGCTCCAACAAAGGTTCTTTTAGTTCCTTTATCTAACCACA**AAGAT**TTAGTT
CCTGTTAC**TACTG**AAGTCGCCAAAATCTTAAGAAAGTCTCAAATC**CCATT**T**AAGAT**T**GATGA**TTCTGGAG
TTT**CCATTGGTA**A**AAGAT**ATGCCCGTAATGACGAATTA**GGTACTCCATTC**GGTGTCA**CCATT**GACTT**TGA**
ATCTGCCAAAGA**CCATTC**TGTCACTTTGAGAGAAAGAGATTCCACCAAGCAAGTTAGAGGTTCCGTCGAA
AATGTCA**TCAAG**GCTATCCGCGATATCACTTAC**AATGGT**GCTTCTTGGGAAGAA**GGTAC**CAAGGATTTGA
CTCCTTTTATTGCCCAAGCTGAAGCTGAAGCTGAAACTGACTAA

38 1.9% 2000 **15.6 5.6σ 10^{-8}**

Archaeoglobus veneficus SNP6, complete genome GenBank: CP002588.1: c173853-172147 Glycyl-tRNA synthetase

ATGAGCGAAATCATGGAAATGCTCATCAGAAGGGGCTTTCTCTGGCAATCCTTCGAAATATACGGAG**GAA**
TGGCCGGATTTATTGATT**ATGGT**CCTCTCGGT**AATGG**ATTGCGTAGGAGGCTCGAGAACCTCTGGCGCGA
GTTCTTCGTAGTTAACGAGAGAGCTGTCGAGATAGACACGCCAACCGTAGGAATAGAGGAAGTTTTCATA
GCCTCCGGCCACGCCACATCCTTCACCGACGTGGCTAT**TGAATGCAAGAA**GTGCGGTAGAGTTTTCAGGG
CCGACCACTACATAAAGGAGAAGCTCGGAATCGAGGCT**GATGA**GACCGTTGAGGCGGTGAAGGAGATCCT
CGAGAGCTTCGACTTAAAGTG**TGAATG**TGACGGTGAGTTTGGAGAACCAACGCCG**TTCAA**CCTCATGTTC

GAAACGAAAATAGGGCCGGGGAAGGGGAAGAAGGGCTACCTGCGCCCTGAAACGGCCCAGGGAATATTCA
TTGCCTTCAAGAGGCTGGCGGACTATTTAGAGATAAGCTTCCCTTTGGAGTTGCCCAGATTGGAAGGGC
TTACAGAAACGAGATAAGCCCGAGACAGGGTGTGATCAGGCTCAGAGAGTTCAATCAGGCCGAACTTGAG
TTCTTCGTCCATCCGGGCGAGAAGAAGCATCCTGATTTTCACATTTATGCAGATGATGAAGTCAAGCTTC
TCGACAAGTTTGGGAACGAGCATGTCATAACTCTCAAAGAGGCTGTTGAGAAGGGAATAATAGCCAATGA
GGTTATAGCGTACTTCATTGGAAAAACGAGGAGATTCCTTCTTCAGGCAGGTATAGACGATGAAAGGTTG
AGGTTCCGGCAGCACCGCGACGATGAAAGGGCTCACTACGCCTCGGACTGCTGGGATGCAGAAGCCTTAA
CGAGCTACGGCTGGATAGAGATCGTAGGAATTGCAGACAGAGGCGACTACGACCTCAGCAGACACATGAA
GTACAGCGGAGAGGACTTGAGTGTCTTCGTACCCTTTAAAGAGCCCGTAAAGGTGAAGAGGAGAAAGATC
GTTCCAAACATGGGCAAGCTCGGGCCGACATTCAGGCAGAAGGCGAAGAAGATTGCCGAAGTTCTTGAAA
GCATGGAGATTGCAGACGATTCTATTGAGGCCATCGAAGTAGAAGTAGATGGAGAGAAAATATCAGTGAG
CAGGGAGTTTTTCGAGGTCAAAGAGGTGGAGGAGCTCATCCACGGTGAGAAGGTTACTCCACACGTCATA
GAGCCCTCTTTCGGTCTCGACAGGATTACCTTTGCTATCCTCGAGCATGCGTTCGACAAAGACATCGTTG
ACGGGGAGGAAAGAAGAGTTCTGAGGCTCAAAAGGTGGATGGCTCCCGTTCAGGTTGCTGTGCTGCCGCT
TCTCTCCAAGGGTGCTTTTATCGAGAAGTCCATCGAGATAACATTCATGCTTAAGAAAGAGGGTATTTTC
ACAGAATACGACGACTCCGGAAGCATAGGAAGGAGGTACAGAAGATTCGACGAAATCGGTACGCCTTTCT
GCGTTACGGTTGACCACCAGACTTTTGAGGACGAGACAGTAACGATAAGGGACAGAGATACAACGAGACA
GATAAGAGTCCATATAGGGGGTCTCGCTCCAGCACTTAGGGAGCTGCTCAGTACAGACAAAGATATATCC
GAGTTTGGAGAGGTCTTTAAAGAATAG

23 1.35% 1703 13.3 (3.6) 2.7 10^{-5}

Table S6. Nucleolin sequences from different species with after the sequence: Nb of observed pentamers among the set {ATTCA TTCAA TCAAG CAAGA AGATG GATGA ATGAA TGAAT} with percentage among the set of pentamers of the sequence (in red), Nb of nucleotides in the sequence (in black), Nb of expected pentamers with its standard deviation (in red), Nb of standard deviations between observed (in blue) and probability of this event (in green).

Bauhinia variegata isolate BV-YZ2020 chromosome 1, whole genome shotgun sequence GenBank: JAKRYI020000001.1: 6791445-6791571,6791692-6791734,6791876-6792039,6792161-6792274,6792411-6792530,6792613-6792729,6792815-6792922,6793010-6793024,6793315-6793389,6793503-6793604,6793720-6793824,6793948-6794052,6794161-6794277,6794388-6794507,6794592-6794672,6794760-6794851,6794924-6795041,6795250-6795338,6795438-6795528,6795666-6795757,6796366-6796655,6796872-6797144 nucleolin

TCCCAAAACCCTAAACTCTGTGACGCGTAGGGTTTTGCAGAGCTTCTCGTTATCTATCCATTTCCTCGAC
CTTTTTCTATTCTTTGTGTCTAATATGGCAAAGTCTAGCAAGAAATCAGCTTCCAAAGTTGAGTCCGCTC
CAGCCGTCGTTCCAAAATCTGCGAAGAAAGGTGCTAAGCGAGAAGCTGAGGATGAGCTTGAGAAGCTGGC
GAGTGCAAAGAAGCAGAAAAGAGATGAGCTTGCTGACAAGCAATTGAATGCGAAGGTGCAAAAGAAAAAG
AAACCGCAAAGTAGTTCATCTGATTCCTCTTCATCCAATTCTGAGGAGGATAAGAAACCTGCTGTTAAAG
TTCCTATTCCTTCAAAGAAGCAACCTGGCCCTGCAAAGAAAAGCAAGCCAGCAAGCAGCTCCAGTTCTGA
CTCTTCTGATGACTCAAGTGAGGATGAAGCTCCAAAATCTAAGGTGAATGGACCTGCCGCGGGTAAGAAT
GGACCTGCTGCTACAACGAAGAAAGTTGATTCTAGTGAAAGCTCTGACTCTGATGACAGCAGCTCGGATG
AAGATAATGCTCCAAAATCCAGGGTGAACGGAGCGGCTGCTGGTAAGAATGGACCTGCTACTACAAAGAA
AAAAGCTGATTCTAGTGAAAGCTCTGATTCAGATGACAGCAGTTCAGATGAAGATTTGGCAGCTGCAGTT
GCTCCTAAGAAGAAAGTGGAAGCAGTTGCTCCAAAGAAAAAAGTTGAGAGCAGTGACAGCTCAGAAGATG
AATCAAGCGCTGAAGAAGATAATGATTCAAAAACTAAGGAAAAGATGGATGTTGATGATGATAGTTCTGA
AGAAAGTGAAGAAGAGCCACAAAAGAAAAATATCAAAGGCTTGCCAAATGCTGGAAAACCTAATGTAAAA
GCTGTCAAAAAGGATAGCAGTAGTGATGGTTCAGAGGATGATGATAGCTCTGAAGAGGATGACAAAGTAA
AAAAGCCAAATGCTGGAAAACCTAATGTAAAAGCTGTTGAAAAGGGTAATAGTAGTGATAGCTCAGATGA
TGAAGATGATAGCTCTGAAGAGGATGAGAAAGTGAAAAAGCCAAATGCTGCAAAACCAAGTGTAAAATCT
GTCAAAAAGGATAGCAGCAGTGATAGCTCAGATGATGAAGATGATAACTCTGAAGAGGATGAGAGAGTGA
AAAAGCCGATTGCCACATCAAGTGCTGGAAAACCTGGTGTAAAAGTTGTCAAAAAGGATAGCAGTAGTGA
TAGCTCAGATGAAGATGATAGCTCTGAAGAGGAGGATGAGATAGTGAAAAAGCCAAAGACTACACCAAAT
GCTGGAAAACCTAGTGCAAAATCTGTCAAAAAGGATAGCAGTAGTGATAGCTCAGATGATGAAGATGATA
GCTCTGAGGAGGATGTAAAATCTGTCAAAAAGGCTAAAGATGTGAAGATGGTTGATGCAGCCTCACCAGA
GCCAACTTCCAAGAATTTTAATTCAGCATCTGCTAAGAAAACTCCTAAGACTCCAGCTACCCCTGTAGAA
CAAAATTCCACAGGAAGGACACTATTTGTTGGAAACTTGTCATATAGTGTGGAAAGAGCTGATGTGGAAA
ATTTCTTCATAGATTGTGGAGAAGTTGTTGATGTCCGCCTTGCTGTAGATGATGAAGGAAGGTTTAAAGG
TTTTGGACATGTTGAGTTTGCTACTGCAGAAGCAGCCCAAAATGCCCTGAAGTTGAATCAAAATGAATTG
TTGAGACGACCCGTCAAACTTGATCTGGCACGAGAAAGGGCCTCATATACCCCTAACAGCAGCAACTGGA
GCAACTCCTTCCAGAAAGGTGGAAGAGGTCAGTCTCAAACAGTATTTGTTAGGGGTTTTGACTCATCCCT
AGATGAACAGCAGATTAGGTCTAATCTGGAGGAGCATTTTAGTTCCTGTGGAGAAATTACTAGGATATCA
ATCCCTAAAGATTATGACACTGGTGCTTTTAAGGGGTATGCATACATGGACTTGAGTGATGCTAATGGCA
TAAACAAAGCATTAGAACTCAATCAATCAGAGTTAGGCGGTTATCCATTGATGGTTGAAGAAGCAAGGCC

TCGGGATTCTCAGGGTGGCAGTGCTAGAGGTGGAAGAGGTGGTAGAGATGGTGGCGGCCGTGGTGGAAGA
GGTGGTAGAGACAGTGGTGGCCGGGGTGGAAGAGGTGATCGTGGAAGAGGTAACCGCGGTGGAAGGGGTG
GCCGTGGAACCCCCTTTAAACAAAGTTTCAGTGCAGGAGGAACAGGGACCAAGACTACTTTCGCAGATGA
CTAGTAGTTCCAAGATTTTGCTGCCTAGATTTTGCTCTAGCGAATATTATTTGTCGTATTTTCTTCTTCT
TTTTAACTCATCAGTTTTGGTAATTTTGATCATTTCACTAAATTCATATGTTTTGGAACAACTATGTTAT
ACTGAGTATCTGTAGCTCTCCAAACACTAGATTGAAGCTACAGTATTATTATGTCTTGTTTAAGTGTTGG
TAGCTTTCGCATTCTATTTTCTTAATATCTTGTCAGAC

78 3% 2554 20 (4.47) 13σ 10^{-30}

Lactobacillus lindneri DSM 20690 = JCM 11027 genome assembly, contig: BP43DRAFT_scaffold00002.2, whole genome shotgun sequence GenBank: FUXS01000002.1: 12949-14616 Nucleolin

TTGATACTTCTTTATCAGTCTTTAGCATTTTCAGTAAGTGCTTCTGAAGGCAGTGTTTCAACTAGTATTT
CAAATTCTGACGAATCAGATAGTGCTAGTGAAAGTACTTCAAATAGTACCTCAAGTAGTATATCTAATTC
TGACGCTTCTACTAGTTCTAGTGAGAGTACCTCAACTAGTTCATCTATAAGTGCTTCAACGAATAAAAGT
GAAAGTGACTCAATCTCTGAAAGTGGATCTGGAACAAACTCACAAAGTACTTCTCAATCAGCTTCAACAA
GTACAAGTGAAAGTGATTCAAGCAATAGCGAATCAAGTTCTGTCAGCGATAGTGACTCAGCCTCCACAAG
TGCATCTGAGAGTGCATCAGCATCAGATAGTACTAGCAATTCAGCAGCGAAATCGGAAAGTAACTCGGCA
GCTAGCGATAGTGAATCAACGAGTGCTTCAAGTTCTAATGCTTCTACAAGTGCTAGTGATAGTGCTTCAA
CGAGTGATTCAACTAGCACCTCAACCAGTACTTCAAGCTCCAATGCCTCGAAGAGTGCGAGCGACAGTGC
TTCCATAAGTACCTCTATCAGTGATTCTACCAAGAAGAGTGATAGTGACTCAGCTTCTATGAGTGGCTCA
GGTACAGATTCAGAAAGTGCCAGCAAGTCAGCTTCCACGAGTGCGAGTGCTAGTGATTCTGCAGCCAAGA
GTACAAGTGATTCAGTAAGTACTTCAGCATCTGGAAAAGACAGTAATGGCTCAACTAGTTCATCAGCTTC
AGCCTCAACGAGTTCATCTGAAGCCAAGAGTACAAGTGATAGTGAATCCACTGTAAATAGTGGATCAGAA
TCAGCAAGTACTTCAGCTTCAAGCTCTGACAGTGCTTCAAGCAGTAAGTCAGCATCAGACCATGGGTCTG
AATCAACGACTGAATCGGCTAGTGCTTCAACCAGTATTTCAGATAGCGAAAGTACTTCGAAGAGTGAATC
AGCAAGTGCTTCAACTAGTAGAGCATCACAGTCAGATAGTGAATCACAAGCTAGTGAAAGTGATACAAGC
AGCAGTGAATCAAGTTCTGTCAGCGATAGTGCGTCAGCTTCCACAAGTGTATCTGATAGTGCATCAGCAT
CAGATAGTACTAGCAATTCGGCAGCAAAATCGGAAAGTAACTCCGAAGCTAGTGATAGTGCTTCAACGAG
TGCTTCTAGTTCCAATGCATCGAAGAGTGCCAGCGTTTCAACTAGTTTCTCAGCTTCAACTTCCACGAGT
GCTAGTGCCTCAGCTTCTATGAGTTTATCTAATAGTGAATTAATGAGTACTTCAAGTTCTAATGATTCAA
CTTCAATTATTGTTTCTGATAGTGCTTCCACTAGTAATTTAAGTTCAAATACTACTAGTACAAACACTAA
CGAATCAGAATCAAAATCTTCTGCAACTATTGCTAGTAGTGGAGTAGTAAATACTTCTGCAACTCAATCA
GCTCAAAATTCATCAGCAAATGGTAATGAATTGAACTCAACAAGTTCTAACAAGTCTGCTAAGAATCAAA
AGACTAATTTACCACAAACTGGACAAGATTCATCAACTGGATTTATGAGTTCTATTGGTTCTATGATTAT
TTCAATCATGGCTCTCTTAGGCCTAGGAAGACTAAGAAAGAAAAAGGAAGATAAATAA

55 3.3% 1664 13 (3.6) 11.7σ 3 10^{-28}

Homo sapiens nucleolin (NCL), mRNA NCBI Reference Sequence: NM_005381.3

AGTCTCGAGCTCTCGCTGGCCTTCGGGTGTACGTGCTCCGGGATCTTCAGCACCCGCGGCCGCCATCGCC
GTCGCTTGGCTTCTTCTGGACTCATCTGCGCCACTTGTCCGCTTCACACTCCGCCGCCATCATGGTGAAG
CTCGCGAAGGCAGGTAAAAATCAAGGTGACCCCAAGAAAATGGCTCCTCCTCCAAAGGAGGTAGAAGAAG
ATAGTGAAGATGAGGAAATGTCAGAAGATGAAGAAGATGATAGCAGTGGAGAAGAGGTCGTCATACCTCA
GAAGAAAGGCAAGAAGGCTGCTGCAACCTCAGCAAAGAAGGTGGTCGTTTCCCCAACAAAAAAGGTTGCA
GTTGCCACACCAGCCAAGAAAGCAGCTGTCACTCCAGGCAAAAAGGCAGCAGCAACACCTGCCAAGAAGA 23
CAGTTACACCAGCCAAAGCAGTTACCACACCTGGCAAGAAGGGAGCCACACCAGGCAAAGCATTGGTAGC
AACTCCTGGTAAGAAGGGTGCTGCCATCCCAGCCAAGGGGGCAAAGAATGGCAAGAATGCCAAGAAGGAA
GACAGTGATGAAGAGGAGGATGATGACAGTGAGGAGGATGAGGAGGATGACGAGGACGAGGATGAGGATG
AAGATGAAATTGAACCAGCAGCGATGAAAGCAGCAGCTGCTGCCCCTGCCTCAGAGGATGAGGACGATGA
GGATGACGAAGATGATGAGGATGACGATGACGATGAGGAAGATGACTCTGAAGAAGAAGCTATGGAGACT
ACACCAGCCAAAGGAAAGAAAGCTGCAAAAGTTGTTCCTGTGAAAGCCAAGAACGTGGCTGAGGATGAAG
ATGAAGAAGAGGATGATGAGGACGAGGATGACGACGACGACGAAGATGATGAAGATGATGATGATGAAGA
TGATGAGGAGGAGGAAGAAGAGGAGGAGGAAGAGCCTGTCAAAGAAGCACCTGGAAAACGAAAGAAGGAA
ATGGCCAAACAGAAAGCAGCTCCTGAAGCCAAGAAACAGAAAGTGGAAGGCACAGAACCGACTACGGCTT
TCAATCTCTTTGTTGGAAACCTAAACTTTAACAAATCTGCTCCTGAATTAAAAACTGGTATCAGCGATGT
TTTTGCTAAAAATGATCTTGCTGTTGTGGATGTCAGAATTGGTATGACTAGGAAATTTGGTTATGTGGAT
TTTGAATCTGCTGAAGACCTGGAGAAAGCGTTGGAACTCACTGGTTTGAAAGTCTTTGGCAATGAAATTA
AACTAGAGAAACCAAAAGGAAAAGACAGTAAGAAAGAGCGAGATGCGAGAACACTTTTGGCTAAAAATCT

CCCTTACAAAGTCACTCAG**GATGAAT**TGAAAGAAGTGTTTG**AAGATG**CTGCGGAGATCAGATTAGTCAGC
AAGGATGGGAAAAGTAAAGGGATTGCTTATAT**TGAAT**TTAAGACAGAAGCTGATGCAGAGAAAACCTTTG 80
AAGAAAAGCAGGGAACAGAGATCGATGGGCGATCTATTTCCCT**GTACTATACTG**GAGAGAAAGGTCAAAA
TCAAGACTATAGAGGTGGAAAGAATAGCACTTGGAGTGG**TGAAT**CAAAAACTCTGGTTTTAAGCAACCTC
TCCTACAGTGCAACAGAAGAAACTCTTCAGGAAGTATTTGAGAAAGCAACTTTTATCAAAGTACCCCAGA
ACCAA**AATGG**CAAATCTAAAGGGTATGCATTTATAGAGTTTGCTT**CATTC**GAAGACGCTAAAGAAGCTTT
AAATTCCTGTAATAAAAGGGAAATTGAGGGCAGAGCAATCAGGCTGGAGTTGCAAGGACCCAGGGGATCA
CCTAA**TGCCA**GAAGCCA**GCCAT**CCAAAACTCTGTTTGTCAAAGGCCTGTCTGAGGATACCACTGAAGAGA
CATTAAAGGAGTCATTTGACGGCTCCGTTCGGGCAAGGATAGT**TACTG**ACCGGGAAACTGGGTCCTCCAA
AGGGTTTGGTTTTGTAGAC**TTCAA**CAGTGAGGAGGA**TGCCA**AAG**CTGCCA**AGGAG**GCCAT**GGAAGACGGT
GAAATTGATGGAAATAAAGTTACCTTGGACTGGGCCAAACCTAAGGGTGAAGGTGGCTTCGGGGGTCGTG
GTGGAGGCAGAGGCGGCTTTGGAGGACGAGGTGG**TGGTA**GAGGAGGCCGAGGAGGATTTGGTGGCAGAGG
CCGGGGAGGCTTTGGAGGGCGAGGAGGCTTCCGAGGAGGCAGAGGAGGAGGAGGTGACCACAAGCCACAA
GGAAAGAAGACGAAGTT**TGAAT**AGCTTCTGTCCCTCTGCTTTCCCTTTT**CCATT**TGAAAGAAAGGACTCT
GGGGTTTT**TACTG**TTACCTGATCAATGACAGAGCCTTCTGAGGA**CATTCCAAGA**CAGTATACAGTCCTGT
GGTCTCCTTGGAAATCCGTCTAGTTAACAT**TTCAAG**GGCAATACCGTGTTGGTTTTGACTGGAT**ATTCA**T
ATAAACTTTTTAAAGAGTTGAGTGATAGAGCTAACCCTTATCTGTAAGTTT**TGAAT**TTATATTGTTTCAT
CCCATGTACAAAA**CCATT**TTTTCCTACAAATAGTTTGGGTTTTGTTGTTGTTTCTTTTTTTTGTTTTGTT
TTTGTTTTTTTTTTTTTTGCGTTCGTGGGGTTGTAAAAGAAAAGAAAGCA**GAATG**TTTTATC**ATGGT**TTT
TGCTTCAGCGGCTTTAGGACAAATTAAAAGTCAACTCTGG**TGCCA**GACGTGTTACTTCCTAAAGAGTGTT
TCCCCTG**GAATG**TCACTGGAGAGCATGGCAAAGCCAGCT**CTGCCA**CTTGCTTCACCCATCCC**AATGG**A**AA**
TGGCTTAGTGCGTGTTTCCAGTATCCCAGCCCTAACTAACTTGGTTGAAATGCTGGTGAGGGGACCTGCT
CCTGCAGCCCTGGTGCTGACTTGAAGGCTGCTGCAGCTTCTCCTACTTTTAGCAGGTCTGAGGATTATGT
CCTGAAGACCACTCTGGAAAGAGGTGCAGGAACAGATTAGTCAGGTTTCCTAGGACAAGGAAGAGCTTCA
GGGAAGAGCAGTGGCTAACTCCTGTAATCCCAACACTTGGGGAGGCCGAGGCAGGCAGATCAACTGAGGT
CAGGAGTTGAAGACCAGCCTGGCCAAC**ATGGT**GAAAGCCCATCTCTACTAAAAATACAAAAATTAGCTGG
GC**ATGGT**GGT**GTACT**CCTGTAGTCCCAGCTACTCAGGAGGCTGAAGCGGGAGAGTCACGTGAACCCGGGA
AGCAGAGTGAGCTGAGCACACACTACTATACTCCAGGCTGGGTAACAAAGCGAGACTCCCATCTCCCAAA
AAGCAGTTCTGGAATAGAACTCACGCT**AGATG**GATAGACCAGTGGACACTTTGGAACCTTGGGGCTGGGG
AGGAA**ACTGCC**CATCCAGTAAACCCCCAAAAA**GCCATT**TGTTCTGCACTACGTATATTGCTTATTCTTTC
TGGTCTTAA**GTACT**TGCCTCTCAACCTCCCTTTTTAGTAAAAGACAAGGCCACGTGAGAGGCGGGACTAT
CAACATTGT**GATGAAT**TTACTTGAAACCCAGTGCCCAAAATCAATGTAGGTAGCCAAGTCCAAAAACCTG
TTCTAGTCCAACTAGTGAAATCAAACTGTGATACTTGGATAAGCTTAGAAGGAAACGTGAAGAATACGTA
GCTGCTTTGGGTTTACTCTGGTTCAGTTGGGCTGTTGAAATCTTAACATCCTTGGGCTTATCACC**TACTG**
CTTGTCAGCCCTGTTCCATGTCCAGGGGATGGGGGTGGTGACAATCCAGTTC**CAAGA**CCCTCATGCTCTA
GAGAGGAAGGTGGCCAGCCAGGGTTGTAACTAC**GATGAA**AAAGCAGTGGGAGGGTCTCCTATGAGGCAAG
CCTAAGGACAAAAAGGAAGGCCTTGCAGCCTGTATTCTGGATAAGGAATTAAAAGCTCAGTTAATTGAAGCCCA

92 2.35% 3920 **30.6 (5.5)** **11.2σ 8 10^{-26}**

PREDICTED: Ornithorhynchus anatinus nucleolin (NCL), mRNA NCBI Reference Sequence: XM_029064618.2

TCAGGCTCGAG**CTGCCA**GCAGGGAGCAGGAGCCCCGACCCGCGTCAGCGCCCGCCGCCCTCCGCCTTTGCCCGAG
CGATCTCT**CTGCC**CTTCCCGCTCGCCCTCTCTCCCTAGGCC**GCCAT**C**ATGGT**GAAGCTCGCGAAGGCAGCTAAAC
AAGGAGGACCGAAGAA**AATGG**CTCCACCTCCAAAGGAGAT**AGATGA**TAGTGAG**GATGAAGAT**ATGTTGGAG**GATG**
let7 3′-CTGTATGGTCT-5′
AAGAGGACAGCAGTGGAGAGGAGGTTGTTATCCCTCAGAAGAAGGCAGTGGCTACACCAGGGAAGAAGGGAG**CTG**
CCG**CTGCCA**CCCCAGGGAAGAAA**GCCAT**GGCCACCCCAGGAAAGAAGGCTGTGGTGTCACCAGC**CAAGA**AGGCCG
TGGTGGCCACCC**CTGCCAAGA**AGGCAGCTGCAACACCGGG**CAAGA**AAGCAGTCGCGGCTGCAGCCACCCCAGC**CA**
AGAAAGCAGTCACCT**CTGCCA**AAGCAGCACCCACCCCAGGGAAAAAGGGGGCCACCCCCGCCAAAGCAGCACCCA
CGCCAGGGAAGAAGGGAGCCACCCCGGCCAAGGGGGC**CAAGA**ACGGAAAGAACGC**CAAGA**AGGCTGAAAGTGAGG
AAG**AAGATGA**GGAAGAGAGTGAAGACAATGACGACTCTGAGACTGAAGCTTTGAAAATAGTTGCACCAAACAAAC
ACGAC**GATGAAT**AC**GATGA**G**GATGATGATGATGA**C**GATGA**C**GATGATGAAGATGA**CTCTGAAGAAGAACCCATGG
AGATCACACCAGCGAAGGGAAAGAAGGCCCCACCTTTGAAGGCTGCTTTGAAAGCCAAGGTGGAGTCTGAGGAAG
AAGAGGAGGAG**GATGAAGATGA**TGGG**GATGA**GGAGGAGGAGGAGGAG**GATGA**GGAGGAACCTGTGAAAGAAACAC
TTGGAAAACGAAAGAAAGA**AATGG**CCAAGCAGAAAGCTGCACCAGAGGCAAAGAAACAGAAAACTGAACCGATTG
AACCTGCATCAGCT**TTCAA**TCTCTTTATTGGAAACC**TGAAT**TTCTCCAAA**ACTGC**TCCAGAGCTGAAAACAGGCC
TCAGTGATTTCTTTAC**CAAGAATG**ACCTCACCGTTCTGGATGTGAGAGTTGGTGTTTCCAGGAAATTTGGCTATG
TGGACTT**TGAAT**CTGCT**GATGA**TCTGGAAAAGGCCCTGGAAT**TGAATGG**CTCTAAGGTCTT**TGGTAATGAAATGA**
AACTGGAGAAAGCAAAGGCAAAAGAGACAAAGAAAGAACG**AGATGCCA**GGACCCTCTTTGTGAAGAATCTTCCCT
ACAAAGTTAC**TCAAGA**AG**AGATGAA**AGAGGTGTTTGAGGATGCTGTTGATGTTAGATTAGTGACTGGCA**AAGATG**

GAATGAGTAAAGGGATTGCCTACATAGAATTTAAAACAGAGGCTGATGCGGATAAGGCCTTGGAAGAGAAGCAGG
GAACTGAAATTGATGGACGTTCAATTATCCTGGACTACACAGGGGAGAAAAGCCAGGGCCTAGAAAACTCCAGAG
GTGGAAAGAACAATGCCTGGAGCGGAGAGTCAAAGACCATTGTCGTGAACAACCTTGCCTACAGTGCAACAGAAG
AAGGTCTCCAGGAAGTTTTTGAAAAGGCCACTTCAATCAAAGTGCCACAGAACCACCAGGGCAGGCCTAAAGGAT
ATGCATTTGTAGAGTTCTCTACTCCTGAAGAAGCTAAAGAGGCTCTAAACTCCTTGAACAATGTAGAAATTGAAG
GTAGAACAATCAGACTGGAGATGCAAGGATCAAAAAATACTAACGCAAGAGGAGGGTTCAGCCACCCATTCAAGA
CTTTGTTTGTCAAAGGCCTGTCTGAAGAAACCACAGAAGAGACGTTGAAAGAGTCATTTGATGGCTCTGTTCGTG
CTAGGATAGTCACTGACCGTGAAACTGGGTCTTCCAAAGGATTTGGCTTTGTGGACTTCAACACCGCAGAAGAAG
CCAAAGCAGCCAAAGAGGCCATGGAAGATGGAGAGATTGATGGAAATAAAGTTACGCTGGACTGGGCCAAGCCCA
AGTCCGAAGGTGGTTTTGGAGGCCGCGGTGGTGGCAGAGGAGGATTTGGTGGCAGAGGCGGTGGCCGGGGCGGTG
GCCGAGGTGGGTTCGGTGGCCGCGGCCGTGGAGGCTTTGGAGGACGGGGAGGCTTCCGAGGAGGCAGAGGCGGCA
GTGGTGGAGACAAGCAACCAGGAAAGAAGACAATTTGAATAGCTTCCCACTCCCCTTTTCCCCCCTCCCTTTCCC
TCTGCTATTTGAAAGGACTCTAGGGTTTTTATTCTGTCATCTTGTCACTGACGGAGCCTTCTGAGGACATTCCAA
GACGACATGCAGTCCTGTTATCCACTTGGAAACATAGATATCATTTCAAGGGAAAGAGAGCGCGCGTCTATTTTG
ACTGCTTGAGTATTCGTATGGACTTTTTAAAAGAGATGAGAGGTAGATTGTGGGAGTTAACTTTGTCTGTGAGTT
GTCTTGAATTTATAATGTTTTACCCCCCATGTACAAAAAATTTTTTTCCTATGAATCTCTGTAGTTTTTTTTGCT
GTAAAAACGCAGAATGTTTTATCATGGTTTTTGCTTCGGCAGCCTGTCTTGGGACAGATTAAAGGGCCTAAACTG
GTGG

70 2625 20.5 (4.5) 11σ 5 10^{-25}

PREDICTED: Acanthochromis polyacanthus nucleolin (LOC110950732), mRNA NCBI Reference Sequence: XM_022193488.2

GAGTAAGGAGCGCGTCTTCGTCTAATCTGTCTTTCTGTCCAATGTCCCACCGAGTAGGCACAGCCACGTGCATGC
AGTAGCTGTTTTGTTACTTGTCTATAAACGTCCAGGTCCCCGTGTTTCACTGAAACACACCACAGCAGCACCATG
GTGAAGTTAGCAAAGGCAGCAAATAATCAAGCAAGTCAAAAGAAAAAGGCACCTCCACCCCCTAAAGAAGTGGAG
GAGGAATCTAGTGAAGAGGACAGCAGCGATGAGGAAGAAGAGGCCCCTCCCCCCAAAGTGGTGAAGAAAGCCACA
CCAGCTAAAGCAACCCCGGTTGTTAAAAATGGTACTGCTGCCAAAAAAGCAGAAAGCGAAGATGATGACGATGAC
GACGACGAGGAAGATGATTCTGAGGAGTCTTCTGAAGAGGAGGCTCCCCCACCTAAGAAGACACCTGCCAAGGCA
ACACCAGCTAAACCTGCAGCAGCTAAGGCAGCCCCCGCAAAGGCAGAGGAATCTGATGATGACGACGATGATGAA
GATGATGATGAGTCTGAAGAAGAGGCCCCACCACCCAAGAAAGCAGCCAAGCCAGCAGCTAAACCTGCCGCCAAG
GCCAAACCTGCAAAGGAGTCTTCTGATGAAGATGATGATGACGACGATGAGGATGATGATTCAGAAGAGGAGGCC
CCACCACCCAAGAAGGCTACTCCAGCTAAACCAGCTGCAAAGCCTGCAGCAAAAGCCAAGCCTGCTAAAGAAGAG
TCGTCTGATGAGGATGATGACGATGAAGAAGATGACTCTGAAGAGGAGATGGACACCACTCCTGCCCCAGCAGTC
GCCAAAGCAAAGAAAGCAGGCATGGTGAAGGCCAAGGAAGAGTCAGAGGAGGAGGATGATGATGATGACGACGAC
GATGATGATGAGGATGACGAAGAAGAAGATGATGCCCCAGTGACACCTGGAAAGCGGAAGGCAGAGACCAAGAAA
GACACACCTCCAGCCAAGAAAGCAAAGTCAGAAGGAGAAGGATTCTGTCTCTTTGTTGGAAACTTGAACTCCAAC
AAAGACTTTGATGAGATCAAAGCATCACTGAGAAAGTTCTTCACCAAGAATGACCTTGAGGTCGTCGATGTCCGG
TTAGGAGGATCAAAAAAGTTTGGTTATGTGGACTTTGGATCTGAAGAGGACCTGCAGAAGGCACTGGAGCTCAAC
GGCAAGAAGATCATGGGTCAGGAGGTCAAACTGGACAGAGCCCGAAGCAAAGAAGGCTCACAGGATGGCAGGAAA
GAGAGGGATGCACGGACACTGTTTGTAAAGAACCTTCCCTTCTCTGCAACGGCTGATGACCTGAAAGAAGTCTTT
GAGGATGCTGTTGACATCAGGCTACCTCAGGGCCAGAACGGTTCAAACAGAGGCATTGCCTACATCGAATTCAAA
ACTGAGGCTGAAGCAGAGAAGATGTTGGAGGAGGCCCAGGGAGCTGATGTACAGGGAAGGTCTATAATGATTGAC
TTTGTTGGAGAAAAGAGCCAGAAAGGAGCCAAAGTGGCAGCATCAGGAGCAGTCAATAAAACGCTGGTAGTGAAT
AATCTTGCATTCAGTGCTACTGAGGAAGTGTTGCAGTCTACATTTGAGAAGGCAGTCTCTATCAGGATTCCACAG
AAAGATGGCAGGCCCAAAGGTTTTGCTTTTATAGAGTTTGAGAGCAATGATGATGCTAAGGAAGCTCTGGAAAAC
CTCAACAACGCAGACATTGAAGGCCGGTCAATCCGACTGGAGTACAGCCAGAACAGCGGTGCAAGAGATGGGGGC
AGGGGAAACTCGGGTCCAACAAAAACCCTCTTTGTCAAGGGTCTGTCTGAGGACACTACAGATCAGACCCTTAAG
GATTCCTTTGAGGGTGCTGTAGCAGCCAGGATAGTTACAGACAGAGACACAGGGTCCTCAAAAGGCTTTGGCTTT
GTGGACTTTGACAACGAGGATGACTGCAAGGCCGCGAAGGAGGCAATGGAGGATGGCGAGATTGATGGCAGCAAA
GTGACCCTGGACTACGCCAAGCCCAAGGGTGAAGGCGGCTTCCGTGGAGGCCGGGGTGGCGGCTTTGGAGGCGGC
TTCGGTGGGCGTGGTGGTGGCAGAGGAGGGCGGGGCGGTTTTGGTGGTCGCGGTGGCTTCGGCGGAAGAGGTGGA
AGAGGTGGTCCTCGTGGAGGTGGACGCGGAGGCGGACGAGGTGGTTTTGGAGGTGGAAGAGGTGGAGGATTTGGA
GCCAAACCCCAGGGGAAGAAAATCAAGTTTGATGACTAAAGCCTTAGTCTGTTTTTTTTTTTTTTTGAATGGAC
TCTGGTTTCATCAATTTTCAGAGCTTTTGGACATTCCAGAAAGCGTCATTGGTATATATTTAACTGTTAATGGGC
ATTTTAGCCCCTTATTTAGACTCCTCAAACCTTCCACATCCTTCTGTGCCAGAATGGTACTTTGACCTTTTCAGC
TTAAGTGCTATGTGTGAGTAATGAATGTCCCGAATGCAAACCTGCACCTCTTGCCCTTGACAGCTTACTTGCTTT
TCTGAAGGAGAAAAATGGAATGGGTCTGGCAAACAAGTTTGCTGTGAAGATATGTTCGTCTAGACATTGTGTAAA
TTTAAAACAGTTTGTATGATTCATTTTACCTTTTGTAAAAAAAAGAAATACACCTGTATCCATGTTTTTTTTGTTT
TTATTTTGTTTGTCATTTAATTTGCTTTAGATGCCAAGTTTTACTGTAATGTGCAAAGCATATTTTGATAGCTTT
GAGCCCATGCTCAACAGAGTTGAGTGTTTAGGATTATGGATGTTTTTGTCTATTCGTTTGGTAGGGCACAGTCAG

TGTTTAAGTTCAGGATGGAAGACCTAATCTATGTAACATAAAATCTTTGTAGTGCATTGACTAATTTTTCTCCCA
TCTC**TCAAGAT**TTTCTCGTAAATAAATAAAACCTTGTAGGTGGTGATTTTTAAA

75 2.5% 2975 **23.2 (4.8) 10.8σ 5 10^{-24}**

Table S7. 5S of Archaea with their AL-pentamer observed (in red), Nb of nucleotides in the sequence (in black), Nb of expected pentamers with its standard deviation (in red) and Nb of standard deviations between observed (in blue).

Methanococcus voltae A00001 5srRNAdb

TGATACGGCGGTCATAGCGGAGGTGTCCCATCCGATC**CCATTC**CGATCTCGGAAATTAAGCCCTCCAGCGA
TTTCTTAA**GTACTGCCATATGGT**GGGAA**CAAGATGA**CG**CTGCC**GATCAC

14 116 **2.5 (1.58) 7.3σ**

Methanococcus voltae A3 strain A3 5S ribosomal RNA, complete sequence NCBI Reference Sequence: NR_075764.1

TAACGGTCATAGCGGAGGTGTACATCCGATC**CCATTC**CGATCTCGGAAATTAAGCCCTCCAGCGATTTCT
TAA**GTACTGCCATATGGT**GGGAA**CAAGATGA**CGCTGTTAGTCA

13 109 **2.34 (1.53) 7σ**

Methanocaldococcus jannaschii DSM 2661 strain DSM 2661 5S ribosomal RNA, complete sequence NCBI Reference Sequence: NR_076076.1

TGGTACGGCGGTCATAGCGGGGGGCCACACCCGAACCCATCCCGAACTCGGAAGTTAAGCCCCCCAGCG
ATGCCCCGA**GTACTGCCAT**CTGGCGGGAAAGGGGCGACACCGCCGGCCAC

8 116 **2.5 (1.58) 3.5σ**

Methanosaeta harundinacea 6Ac strain 6Ac 5S ribosomal RNA, complete sequence NCBI Reference Sequence: NR_103229.1 5S

CGAGTGTGGCG**GCCAT**AGCGGCGGGGCAACTCCTGAAC**CCATTC**CGAACTCAG**AAGAT**AAGCCCGCGTGC
GTTCCA**TACTGTACT**TAGGTGCGCGAGCCCTCGGGAACTATGGATCG**CTGCCA**

8 119 **2.55 (1.6) 3.4σ**

Methanosarcina mazei 5S ribosomal RNA gene, complete sequence NCBI Reference Sequence: NR_075125.1

AGTTTGGCG**GCCAT**AGCGGCAGGGCAACTCCTGTACCCATCCCGAACACAG**AAGAT**AAGC**CTGCC**CGCGT
TCCT**TACTGTACTG**AAGTGTGCGAGCCTTCGGGAACTCTGGATCG**CTGCCA**AGCTCACCT

8 126 **2.7 (1.65) 3.2σ**

Nanoarchaeum equitans Kin4-M 5S ribosomal RNA, complete sequence NCBI Reference Sequence: NR_103265.1

GGAGG**GCCAT**AGCGGCCCGGGAACCACCCGTACCCATCTCGAACACGGAAGTTAAGCCGGGCCGCGTCCC
GAG**TGGTACTGCC**CCGCGAAGGGGTGGGAAGCTCGGGATGCCCTCCC

7 113 **2.4 (1.56) 3σ**

Methanosarcina barkeri str. Fusaro strain Fusaro 5S ribosomal RNA, complete sequence NCBI Reference Sequence: NR_075338.1

AGAGTTTGGCG**GCCAT**AGCGGCAGTGTAACTCCTGTACCCATCCCGAACACAGTAGATAAGC**CTGCC**CGC
GTTCCT**TACTGTACTG**AAGTGTGCGAGCCTTCGGGAACTCTGGATCG**CTGCCA**TACTCACC

7 127 **2.7 (1.65) 2.6σ**

Thermococcus kodakarensis KOD1 strain KOD1 5S ribosomal RNA, complete sequence NCBI Reference Sequence: NR_075222.1

GGTACGGCG**GCCAT**AGCGGCGGGGCCACACCCGGTCTCGTTTCGACCCCGGAAGTTAAGCCCGCCAGCGT
TCCCGGGT**GTACTGCC**CTCCGAGAGGGGGCGGGAAACCGGGAACGCCGCCGGCCAC

6 122 **2.6 (1.6)** 2.1σ

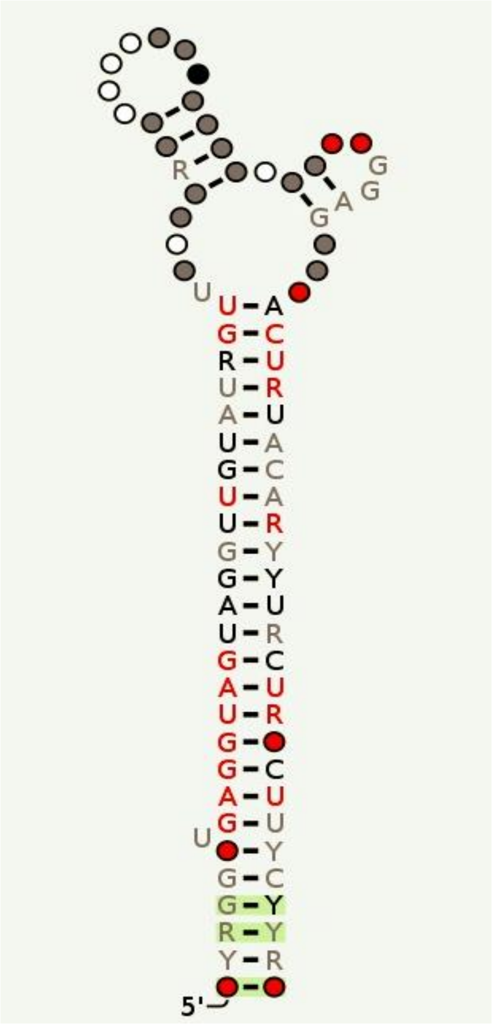

Figure S1. MicroRNA let7 secondary structure
